# Improved Centile Estimation by Transformation And/Or Adaptive Smoothing of the Explanatory Variable

**DOI:** 10.1002/sim.70414

**Published:** 2026-02-05

**Authors:** R. A. Rigby, D. M. Stasinopoulos, T. J. Cole

**Affiliations:** ^1^ School of Computing and Mathematical Sciences University of Greenwich UK; ^2^ UCL Great Ormond Street Institute of Child Health UK

**Keywords:** centile smoothing, distributional regression models, GAMLSS, growth reference charts, LMS method

## Abstract

A popular approach to growth reference centile estimation is the LMS (Lambda‐Mu‐Sigma) method, which assumes a parametric distribution for response variable Y and fits the location, scale and shape parameters of the distribution of Y as smooth functions of explanatory variable X. This article provides two methods, transformation and adaptive smoothing, for improving the centile estimation when there is high curvature (i.e., rapid change in slope) with respect to X in one or more of the Y distribution parameters. In general, high curvature is reduced (i.e., attenuated or dampened) by smoothing. In the first method, X is transformed to variable T to reduce this high curvature, and the Y distribution parameters are fitted as smooth functions of T. Three different transformations of X are described. In the second method, the Y distribution parameters are adaptively smoothed against X by allowing the smoothing parameter itself to vary continuously with Y. Simulations are used to compare the performance of the two methods. Three examples show how the process can lead to substantially smoother and better fitting centiles.

**Abbreviations**
GAMLSS: generalized additive models for location scale and shape
LMS: lambda‐mu‐sigma

## 1 Introduction

Time‐specific reference ranges are widely used in medical research, notably in the form of growth reference centile charts [1]. The $100p^{\text{th}}$ centile (or $p^{\text{th}}$ quantile) of a continuous response variable Y (e.g., height) conditional on value x of explanatory variable X (typically age) is denoted $y_{p}(x)$ and defined formally by $P(Y\leq y_{p}(x)|X=x)=p$, where 0<p<1. A growth chart consists of a set of smooth centile curves $y_{p}(x)$ defining the time‐varying distribution of Y. The LMS method is a popular approach to centile estimation [2] that assumes a parametric distribution for Y and estimates its distribution parameters as smooth curves in X—this ensures that the associated centile curves $y_{p}(x)$ are also smooth. The LMS method fits a Y distribution with three time‐varying parameters:

- L or λ, the shape or skewness of the distribution denoted by λ(x),
- M or μ, the location or median of the distribution denoted by μ(x),
- S or σ, the scale or coefficient of variation of the distribution denoted by σ(x).

The LMS method is a special case of a broader class of models called GAMLSS (Generalized Additive Models for Location, Scale, and Shape), [3] which are fitted in R using the **gamlss** package [4].

The **gamlss** fitting process involves jointly estimating and smoothing the location, scale and shape curves μ(x), σ(x) and λ(x). The smoothing can be done in many different ways ranging from simple linear regression through polynomials and fractional polynomials [5] to variants of cubic splines [6]. Cubic splines provide a more flexible solution than simpler methods, but they can be complicated to fit because the associated knot positions and/or smoothing parameter need to be specified. **gamlss** offers a popular smoother pb() called penalised B‐splines or P‐splines, [7] which is convenient as it sets the knot positions and estimates the smoothing parameter automatically.

**FIGURE 1:**
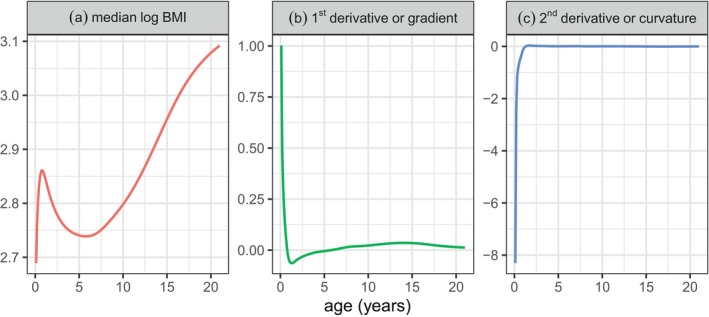
Plots of log median BMI versus age and its first and second derivatives, reflecting the gradient and curvature of the median curve.

However, even P‐splines are not foolproof, and in certain situations they lead to biased curves. This arises when one or more of the parameter curves, typically the location curve μ(x), has high curvature where the gradient is changing rapidly with X (e.g., age). Figure 1 shows median body mass index (BMI) by age in Dutch boys, based on data described in Section 5. The median curve is modelled on the log scale, as shown in Figure 1a, whereas Figure 1b,c shows respectively the first derivative or gradient, and the second derivative or curvature, of the curve. The median increases steeply from birth to a peak at around 9 months and then falls (Figure 1a), during which time the gradient falls steeply (Figure 1b). This corresponds to very high negative curvature for the first 15 months of life (Figure 1c). During this period the P‐spline can reduce (i.e., attenuate or dampen) the curvature leading to biased centiles.

There are two ways to minimise the bias: either transform the X scale so as to stretch it in regions of high curvature, and hence reduce the curvature, or use a smoothing process that is more sensitive—that is, adapts—to changes in gradient with X.

In this article, we aim to improve centile estimation of Y by better handling high curvature, either by transformation of X [8, 9] or by adaptive smoothing [10]. Three different transformations are considered: a shifted power transform of X; a signed shifted power transform of the absolute distance of X from a specific interior value $x_{0}$, or (restricted to monotonic μ(x)) a transformation of X using an initial smooth of μ(x). In all three cases, the Y centiles are estimated on the transformed X scale and plotted on the original X scale. The other alternative, adaptive smoothing, allows the P‐spline smoothing parameter to vary continuously with X so that the smoothing process is more sensitive to changes in gradient. The smoothing parameter adapts to the varying curvature, decreasing when the curvature is high, and increasing when the curvature is low.

Section 2 gives details about the LMS method and extensions, Section 3 explains the three transformations, and Section 4 describes P‐splines and adaptive smoothing. Section 5 works through three examples, and Section 6 uses simulation to compare the performance of the two approaches. Conclusions are given in Section 7.

## 2 The LMS Method and GAMLSS for Centile Estimation

The conditional distribution of Y given X=x corresponding to the LMS method [2] is represented in GAMLSS [3] as the three parameter Box‐Cox Cole and Green distribution, BCCGo(μ,σ,ν), where the λ of the LMS method is renamed ν. The LMS model is given by:

(1) $$\begin{array}{ll} Y & ∼BCCGo(\mu ,\sigma ,\nu )\\ \log(\mu ) & =s_{1}(x)\\ \log(\sigma ) & =s_{2}(x)\\ \nu & =s_{3}(x) \end{array}$$

where μ>0, σ>0 and −∞<ν<∞, the parameter predictors are log(μ), log(σ), and ν, and $s_{1}$, $s_{2}$ and $s_{3}$ are smooth functions.

Similarly the LMSP (LMS‐Power) and LMST (LMS‐*t*) methods [11, 12] model Y using the four parameter Box‐Cox power exponential, BCPEo(μ,σ,ν,τ), and Box‐Cox *t*, BCTo(μ,σ,ν,τ), distributions, respectively, where the additional parameter τ>0 represents a kurtosis parameter for Y, and its default predictor is log(τ). The LMS, LMSP, and LMST methods of centile estimation are explained in chapter 13 of [13], while the BCCGo, BCPEo and BCTo distributions are explained in Chapter 19 of [14]. All are implemented in the **gamlss** R package [4].

More generally Y can be modelled using any parametric distribution where all its parameters are constrained to vary smoothly with X, see for example [15] where bounded distributions are fitted.

## 3 Three Transformation Methods for the Explanatory Variable

Here we describe three ways to transform X to improve the centile estimation of Y. The first two involve a power parameter λ and/or a shift parameter α, for which optimal values are obtained using the R function optim(). The third method involves fitting two successive smooth curves rather than one, with the predicted values from the first fit used as explanatory variable for the second fit [9].

### 3.1 Shifted Power Transform of X

**FIGURE 2:**
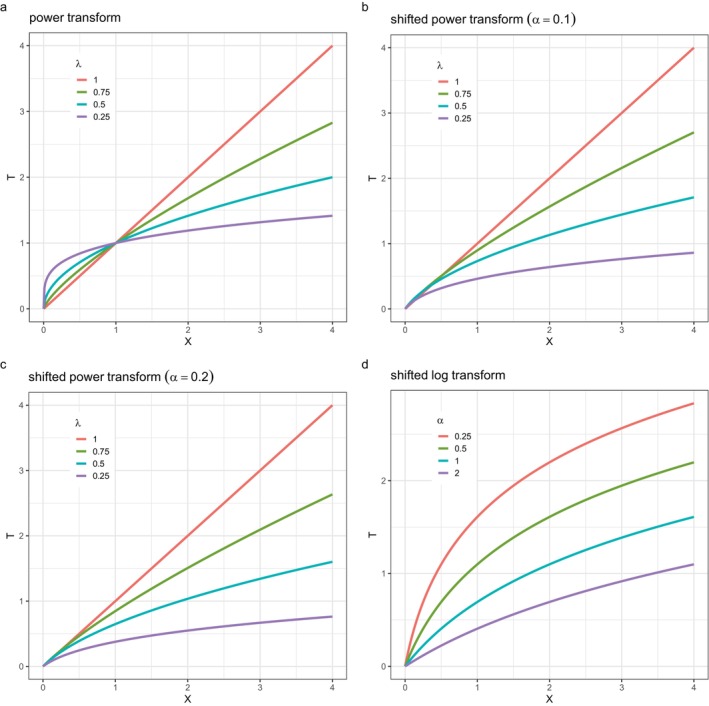
Transformations from X to T: (a) power transform, (b) shifted power transform (α=0.1), (c) shifted power transform (α=0.2), and (d) shifted log transform.

A *shifted power* transform of X is given by

(2) $$T=\left\{\begin{array}{ll} {\left(\alpha +X\right)}^{\lambda }-\alpha^{\lambda },\; & \text{for}\;\lambda >0\;and\;\alpha \geq -min(X)\\ \log\left(1+\frac{X}{\alpha }\right),\; & \text{for}\;\lambda =0\;and\;\alpha >-min(X) \end{array}\right.$$

where α is the shift parameter and λ is the power parameter, and T=0 when X=0. Note that negative λ is not considered here. If α=0, (2) simplifies to the *power* transform of X given by $T=X^{\lambda }$. Figure 2a shows the power transform for λ={0.25,0.5,0.75,1}, while Figure 2b,c shows the analogous shifted power transforms (2) for α=0.1 and α=0.2.

The *shifted log* transform of X is a special case of (2) when λ=0 (since $(X^{\lambda }-1)/\lambda \to \log X$ as λ→0). Figure 2d shows the shifted log transform for α={0.25,0.5,1,2}.

The main differences between the transformations in Figure 2 lie in the gradient and curvature of the curves for X close to 0. The gradient at X=0 is infinite in Figure 2a, for 0<λ<1, but finite elsewhere in Figure 2. The nonlinear curves respectively expand and contract the scale for low and high values of X, and so are well suited to situations where there is high curvature in Y for X close to 0, for example, median BMI in Figure 1.

### 3.2 Signed Shifted Power Transform of the Absolute Distance of X From an Interior Value

A *signed shifted power* transform of the absolute distance of X from an interior value $x_{0}$ is given by

(3) $$\begin{array}{c} T=\left\{\begin{array}{l} x_{0}+\text{sign}(X-x_{0})\times \left[{\left(\alpha +|X-x_{0}|\right)}^{\lambda }-\alpha^{\lambda }\right],\\ \;\text{for}\;\lambda >0\;and\;\alpha \geq 0\\ x_{0}+\text{sign}(X-x_{0})\times \log\left(1+\frac{\left|X-x_{0}\right|}{\alpha }\right),\\ \;\text{for}\;\lambda =0\;and\;\alpha >0 \end{array}\right. \end{array}$$

where $min(X)\leq x_{0}\leq max(X)$ and $T=x_{0}$ when $X=x_{0}$.

**FIGURE 3:**
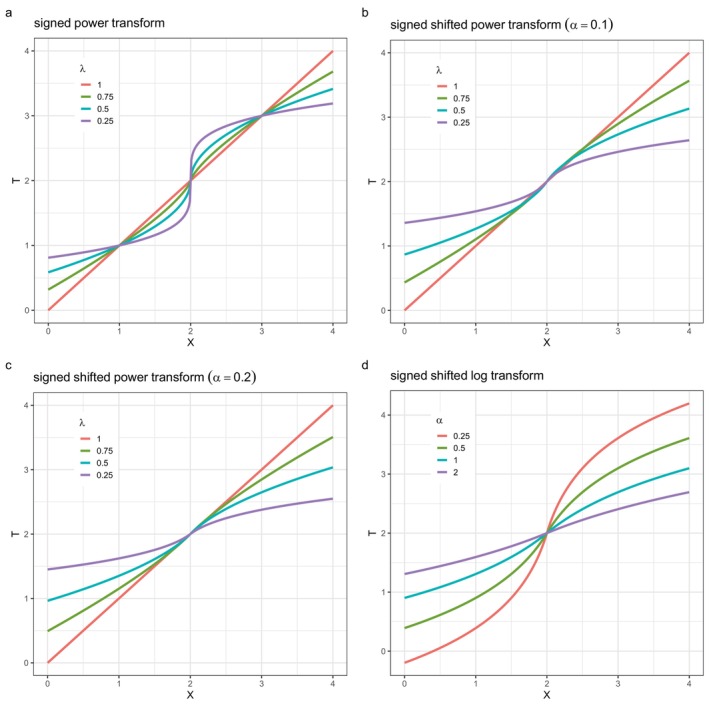
Transformations from X to T with $x_{0}=2$: (a) signed power transform, (b) signed shifted power transform (α=0.1), (c) signed shifted power transform (α=0.2), (b) signed shifted log transform, of the absolute distance of X from an interior value $x_{0}=2$.

Figure 3a shows (3) as a *signed power* transform when α=0, for λ={0.25,0.5,0.75,1} and $x_{0}=2$. Changing $x_{0}$ merely shifts the plot horizontally. Similarly, Figure 3b,c shows (3) as signed shifted power transforms for α=0.1 and α=0.2. A possible disadvantage of (3) when α=0 is that its first derivative at $X=x_{0}$ is infinite, for 0<λ<1, which can lead to a spike in the Y centiles at $X=x_{0}$.

A *signed shifted log* transform of the absolute distance of X from $x_{0}$ is a special case of (3) when λ=0. Figure 3d shows the transform for α={0.25,0.5,1,2}.

Note that the transformations in Figure 3 are effectively those in Figure 2 shifted to origin $\left(x_{0},x_{0}\right)$ and combined with their 180° rotations about the origin.

The nonlinear curves in Figure 3 expand the X scale near $X=x_{0}$ and contract it for X further away from $X=x_{0}$, and so are well suited to situations in which there is high curvature around $x_{0}$, for example, a maximum or minimum turning point in μ(x)—see the example in Section 5.1.

An alternative transformation which has finite first and second derivatives at $X=x_{0}$ is given by

(4) $$T=x_{0}+\sinh^{-1}\left[\alpha \times (X-x_{0})\right],\text{for}\;\alpha >0$$

where $\sinh^{-1}(x)=\log\left[x+{\left(x^{2}+1\right)}^{1/2}\right]$ and $T=x_{0}$ when $X=x_{0}$. (4) gives very similar results to (3) in the example in Section 5.1.

### 3.3 Cole Transformation

The third transform is from Cole et al. [9]. The Cole transformation first fits $T=\hat{\mu }(x)$ the median of Y against X=x. Then it views the explanatory variable as T rather than X, and refits Y against T. On this new explanatory variable scale the fitted predictor of μ is linear in T, and the true predictor is likely to be closer to linear in T than in X due to the first fit reducing the curvature. Then the LMS method is used to estimate the centiles of Y plotted against T, and finally the centiles are plotted against X.

Note that $\hat{\mu }(x)$ must be monotonic in x for the transformation to work properly. We use the transformation to fit height centiles in Section 5.3. It is well suited to situations in which $\hat{\mu }(x)$ has high curvature in more than one region of the X range, for example rapid growth in infancy and puberty.

## 4 P‐Splines and Adaptive Smoothing

### 4.1 P‐Splines

A P‐spline is a form of B‐spline proposed by [7] that uses a penalized smooth function of X to fit Y. The P‐spline minimizes the sum of two terms: a measure of the ‘lack‐of‐fit’ of the function (squared residuals), and a ‘roughness’ penalty (differences between adjacent B‐spline coefficients) that is multiplied by a smoothing parameter λ to control the smoothness of the function. (Note that this λ is different from the power λ in Section 3.) The larger λ is the smoother the function, but at the cost of a poorer fit. Conversely if λ is smaller the function is rougher but the fit is better.

In **gamlss**, the function pb(x) fits a P‐spline and estimates λ automatically. Its choice represents a compromise between goodness‐of‐fit and smoothness, and most of the time the default works well. And if it does not, λ can be varied by changing the effective degrees of freedom of the smoother, for example, pb(x, df = 6). However, when the distribution parameter has sufficiently high curvature this may not be sufficient, resulting in a relatively poor fit *and/or* a rough fitted function, whatever the value of λ. Hence, P‐splines where λ is global, that is, constant across the range of X, can be too restrictive when curvature is high.

### 4.2 Adaptive Smoothing

Adaptive smoothing is a modified form of P‐spline where λ is allowed to vary continuously with X. It is implemented here using the method of Separation of Overlapping Precision (SOP) matrices [10] and fitted in **gamlss** with the function ad(x) from the **SOP** R package [16]. The function ad() has two arguments: nseg the number of segments in X used for the smoothing function, and nseg.sp the number of segments used for the smoothing parameter (see Supporting Information B for details).

In general, the flexibility of a fitted smooth function is increased strongly by increasing nseg.sp, and less strongly by increasing nseg. Choosing nseg.sp is therefore particularly important. The value of nseg.sp needs to be sufficiently large to allow flexibility in the smoothing parameter curve, but not too large potentially causing overfitting. In the analysis in Section 5, we adaptively smooth all the predictors of the Y distribution parameters against X, using the same nseg and the same nseg.sp for all the distribution parameters. Two values of nseg.sp were chosen in each case. First, by searching over integer values from 1 to 5, and then over values from 1 to 10, for the value of nseg.sp giving the minimum value of criterion GAIC(4), (defined in Section 5). The reason for choosing two values of nseg.sp was to find a reasonable compromise between flexibility (avoiding underfitting) and avoiding overfitting.

## 5 Examples

The **gamlss** R package was used to fit the models. The response variable distribution, transformation parameter, and number of segments nseg.sp in the adaptive smoothing were each selected by minimizing the value of the generalized Akaike information criterion GAIC(κ) defined by:

(5) GAIC(κ)=−2l+κ×df

where l is the model's fitted log likelihood, df is its effective degrees of freedom (i.e., number of parameters) and κ is the penalty per degree of freedom. Hence GAIC(0) is the global deviance (DEV), GAIC(2) is the Akaike information criterion (AIC), and GAIC(log(n)) is the Bayesian information criterion (BIC) where n is the sample size. The model with the lowest value of GAIC(κ) for given κ is selected as the ‘best’ model. Often AIC selects a model that overfits the response variable, while BIC can underfit. A compromise is GAIC(4) with penalty κ=4, which approximates the Chi‐squared test statistic $\chi_{1,0.05}^{2}=3.84$.

### 5.1 Triceps Skinfold in Gambian Females

We used data from an anthropometry survey of 892 girls and women aged 0–50 years from three Gambian villages in West Africa, with the variables triceps (triceps skinfold in mm) and age (in years). The data were previously analysed by [2] and are available in the data frame triceps in the **gamlss.data** R package.

**FIGURE 4:**
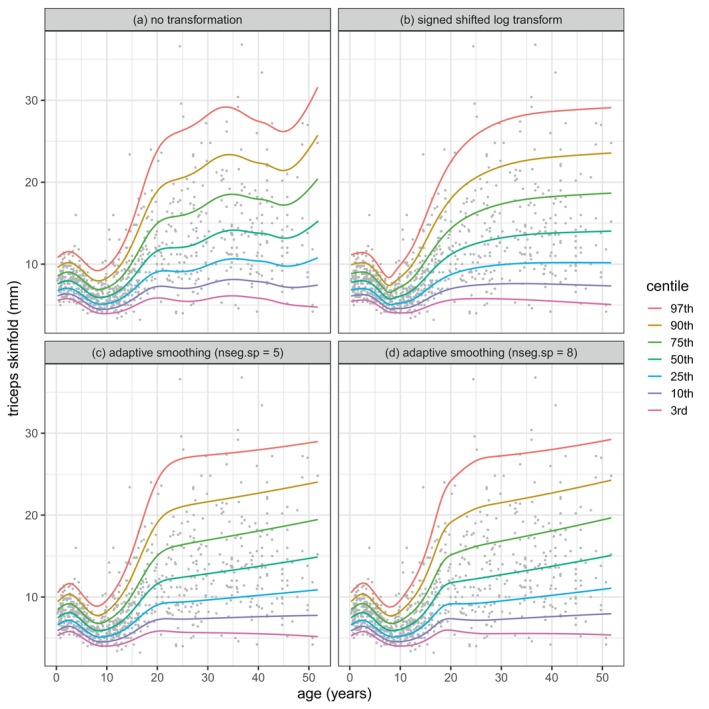
Centiles of triceps against age for the four fitted models for triceps: (a) M1: no transformation, (b) M2: signed shifted log transform, (c) M3: adaptive smoothing with nseg.sp=5, (d) M4: adaptive smoothing with nseg.sp=8.

Initially triceps was modelled using each of the distributions BCCGo(μ,σ,ν), BCTo(μ,σ,ν,τ) and BCPEo(μ,σ,ν,τ), with P‐spline smoothers in age for the distribution parameter predictors. The distribution minimising GAIC(4) was BCCGo. The centiles of the resulting model M1 are plotted in Figure 4a and are clearly not smooth.

**FIGURE 5:**
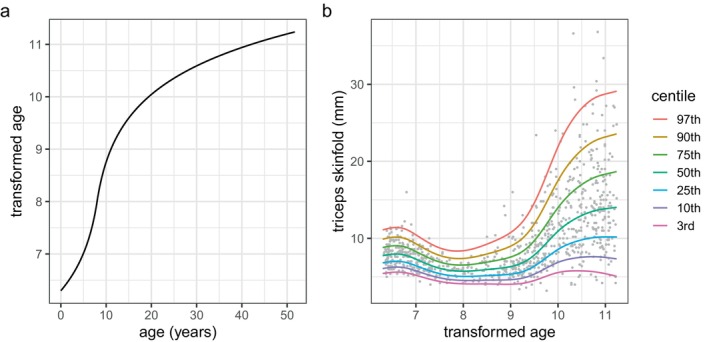
triceps fitted model M2 (a) signed shifted log transform t of age plotted against age, (b) triceps centiles plotted against t, the transformed age.

The fitted median of triceps had a minimum turning point at age=8.0. So next we applied transformation (3), a signed shifted log transform of the absolute distance of age from 8, including the scale parameter α, given by t=8+sign(age−8)×log(1+|age−8|/α). We then used R function optim() to optimize α by minimizing GAIC(4) for each of BCCGo, BCTo and BCPEo. The best model M2 was BCCGo with α=1.78. The centiles of M2 are plotted in Figure 4b and are much smoother than in Figure 4a. Figure 5a shows a plot of transformed age t versus age, and Figure 5b plots the centiles of triceps against t as fitted by M2. The effect of the transformation is to stretch the age scale near age 8 and shrink it further away from 8.

Next we used the adaptive smoother ad() in age with nseg=20 and the optimal value (searching over values from 1 to 5) nseg.sp=5 for all three BCCGo parameter predictors. The centiles of the resulting model M3 are plotted in Figure 4c. We then used the adaptive smoother ad() in age with nseg=20 and the optimal value (searching over values from 1 to 10) nseg.sp=8 for all the BCCGo parameter predictors. The centiles of the resulting model M4 are plotted in Figure 4d. The R code for models M1 to M4 is given in Supporting Information A.

The adequacy of each of the four models was investigated using Z and Q statistics (see [17] and [13], section 12.6) to test the normality of, and worm plots [18] to visualise, their residuals split into different age ranges. See Supporting Information A for the resulting Z and Q statistics. The models M2, M3, and M4 have similar Z and Q statistics and indicate generally adequate fit.

The simulations in Section 6 (see later) show that the GAIC information criteria are inappropriate for comparing the transformation and adaptive smoothing models. Instead we used cross‐validation, splitting the 892 cases randomly into training and test data sets with probabilities 0.7 and 0.3, respectively. We fitted the four models to the training data (including optimising λ amd α in M2 and nseg.sp in M3, and M4), and then calculated their global deviances as applied to the test data. We repeated this exercise 100 times and summarised the fit as the median test global deviance over the 100 values for each of the four models.

Table 1 compares the global deviance DEV, together with the degrees of freedom df (i.e., the sum of the μ, σ and ν parameter degrees of freedom $df_{\mu }$, $df_{\sigma }$ and $df_{\nu }$, respectively), and the median test global deviance TDEV, for the four models. The minimum values of df, DEV and TDEV, subtracted from their columns to make the table clearer, are also shown.

**TABLE 1:** Comparing the global deviance (DEV) and the total (df) and individual parameter degrees of freedom ($df_{\mu }$, $df_{\sigma }$, $df_{\nu }$), and the median test global deviance (TDEV), for the four triceps models M1 to M4.

	df	DEV	$df_{\mu }$	$df_{\sigma }$	$df_{\nu }$	TDEV
M1	3.5	5.0	10.9	5.1	2.0	8.2
M2	2.0	**0**	8.7	4.6	3.1	**0**
M3	0.2	4.0	7.5	4.8	2.5	6.5
M4	**0**	1.4	7.3	4.8	2.5	7.7
Minimum subtracted	14.6	4107.7				1240.7

Table 1 shows that both the global deviance and the test global deviance are smallest for model M2. Hence the signed shifted log transform is effective at providing smooth and well fitting centiles when the data involve a minimum or maximum turning point at an interior value. This has not previously been shown.

### 5.2 BMI in Dutch Boys

The Fourth Dutch Growth Study [19, 20] was a cross‐sectional study of the anthropometry of Dutch children aged 0–22 years. The data used here are bmi (body mass index) and age (in years) in 7294 boys. They were previously analysed by [18] and [11] and are available in the data frame dbbmi in the **gamlss.data** R package. The R code for this analysis is given in Supporting Information B, together with plots of the fitted median, 3rd and 97th centiles of bmi against age under the different models, with 95% confidence intervals based on 1000 bootstrap samples.

**FIGURE 6:**
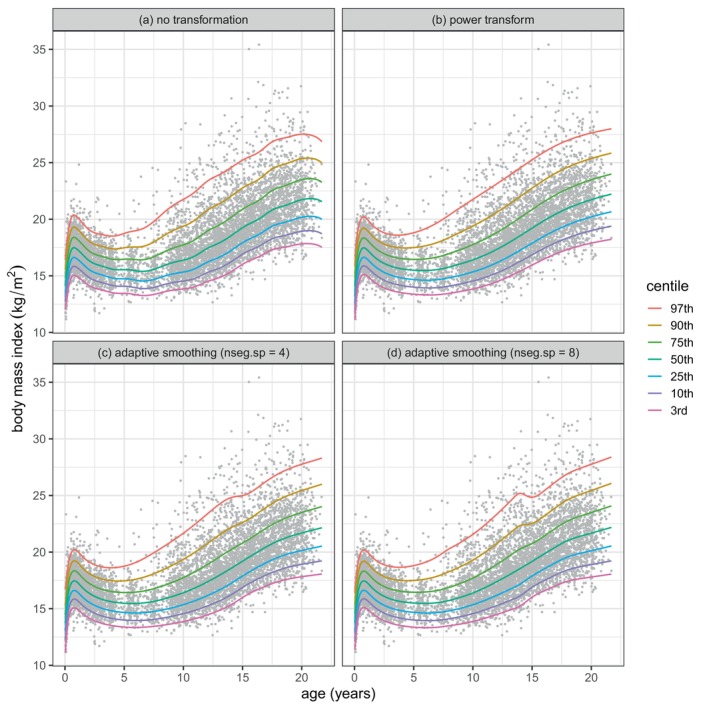
Centiles of bmi against age for the four fitted models for bmi: (a) m1: no transformation, (b) m2: power transform, (c) m3: adaptive smoothing with nseg.sp=4, (d) m4: adaptive smoothing with nseg.sp=8.

Initially bmi was modelled using the approach in Section 5.1 where the distribution minimising GAIC(4) was BCTo. The centiles of the resulting model m1 are plotted in Figure 6a. They are clearly not smooth.

**FIGURE 7:**
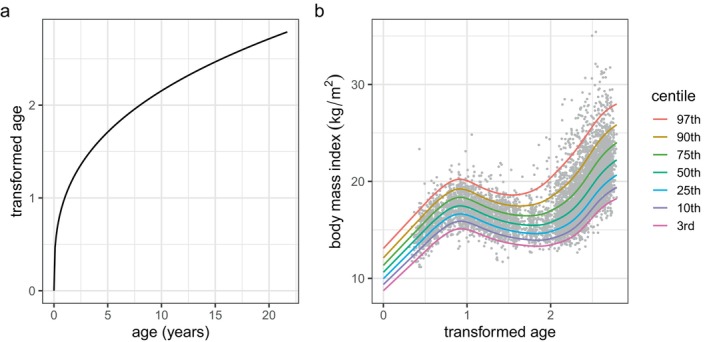
bmi fitted model m2 (a) power transformed age, t, plotted against age, (b) centiles of bmi plotted against t, the transformed age.

Next we applied a power transform $t=age^{\lambda }$ to age and used the R function optim() to optimize λ by minimizing GAIC(4) for each of the distributions BCCGo, BCTo and BCPEo. The best model was BCTo with λ=0.36, and since this was close to a cube root transformation we used λ=1/3, and the resulting model m2 replaced age in m1 with $t=age^{1/3}$. The centiles for m2 are smoother than for m1, see Figure 6b, and they fit better as judged by the global deviance (see Table 2); m2 also uses fewer degrees of freedom than m1 indicating that it is less complex. Figure 7a shows a plot of transformed age t versus age, and Figure 7b plots the centiles of bmi against t as fitted by m2. The transformation stretches the age scale at young ages and shrinks it at older ages.

**TABLE 2:** Comparing the global deviance (DEV) and the total (df) and individual parameter degrees of freedom ($df_{\mu }$, $df_{\sigma }$, $df_{\nu }$, $df_{\tau }$), and the median test global deviance (TDEV), for the four bmi models m1 to m4.

	df	DEV	$df_{\mu }$	$df_{\sigma }$	$df_{\nu }$	$df_{\tau }$	TDEV
m1	12.9	14.2	21.8	6.7	5.4	2.0	7.4
m2	3.4	7.1	12.6	6.3	5.5	2.0	**0**
m3	2.0	3.5	12.7	6.0	3.5	2.9	1.5
m4	**0**	**0**	12.1	6.0	3.0	2.0	3.4
Minimum subtracted	23.1	29 139.3					8757.6

Next we used the adaptive smoother ad() in age with nseg=50 and nseg.sp set the same for all four BCTo parameter predictors. (Note that nseg was increased from 20 as used for the triceps data to 50 for the bmi data due to the larger sample size.) The value of nseg.sp was chosen to minimise GAIC(4), by searching first over integers 1 to 5 and then over integers 1 to 10. There was a local minimum at nseg.sp=4, giving model m3, and the global minimum at nseg.sp=8 led to model m4. The resulting centiles are plotted in Figure 6c,d, and show a feature not present in Figure 6a,b (specifically a levelling off or local peak in the 97th centile at age 14, respectively). The peak is prominent in Figure 6d, reflecting the fact that higher values of nseg.sp generally give more flexible but potentially overfitting centiles, compared to lower values. There are also local troughs in the 3rd centile at age 14, though they are less obvious.

**FIGURE 8:**
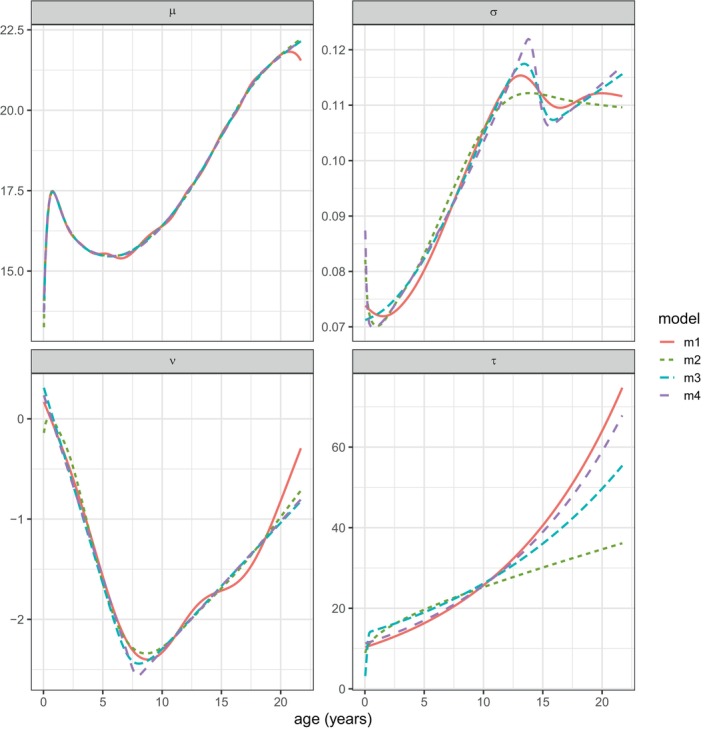
bmi fitted models: the fitted parameters μ, σ, ν and τ plotted against age for the four models, m1, m2, m3, and m4.

To investigate which distribution parameters cause the local peak, Figure 8 shows the fitted parameter predictors for μ, σ, ν, and τ for the four models plotted against age. The curves for μ and ν are very similar at all ages, whereas those for τ and particularly σ differ after age 11. The σ curves all peak at around age 14, but the peak is higher for m4 and m3 than for m1 and m2. It is this peak on the σ curves for m4 and m3 that generates the peak on the 97th centile and the trough on the 3rd centile, by expanding the distribution at this age—the 3rd centile trough is relatively small due to the marked negative skewness at age 14 (where ν=−1.8).

A key question is whether or not the peak in the adaptively smoothed σ curves is genuine, or whether the curves are overfitted and undersmoothed. The peak in the σ curve at age 14 is also seen, though to a lesser extent, with the untransformed model m1, which supports the idea that the local changes in the variance are genuine. It is striking though that the adaptively smoothed predictors for σ and ν are close to piecewise linear, suggesting that the smoothing process somehow “saves” degrees of freedom by linearising the curve and hence exaggerating any turning points. Note that high curvature turning points such as these are in conflict with the original LMS principle that the distribution parameter curves should be smooth.

The pubertal growth spurt is a possible biological explanation for the peak followed by a trough in the σ curve. It is well known that for height and weight the σ curve has a marked single peak centered around age 14 in boys, due to variability in the timing of the growth spurt which is maximal at the mean age of peak velocity [9, 21, 22]. However for BMI, defined as $weight/height^{2}$, the σ peak is largely absent, see model m2 in Figure 8 [9], because the height peak cancels out the weight peak – the variability in the two measurements rises and falls in tandem with age. It is possible that the adaptively smoothed σ curve detects a vestigial pubertal rise and fall in variability followed by a second post‐pubertal rise due to variability in weight gain leading into adulthood.

As in Section 5.1, the adequacy of the models was investigated using Z and Q statistics [17] to test the normality of, and worm plots [18] to visualise, their residuals split into different age ranges. See Supporting Information B for the resulting Z and Q statistics. Only one Q statistic was marginally significant (p=0.03)—the variance of the residuals in model m2 was significantly low in the age range 15.1–16.3, indicating that its fitted σ was too high at that age. This accords with the σ curves in Figure 8.

As in Section 5.1, we used cross‐validation to compare the models, randomly splitting the data into training and test sets 100 times and finding the median test global deviance. In the 100 splits, the lowest test deviance occurred 6, 46, 20 and 28 times respectively for the models m1 to m4, while m4 consistently had the lowest GAIC(4). This confirmed that GAIC(4) was inappropriate for comparing the transformation and adaptive smoothing models.

Table 2 compares the four models in the same format as Table 1. According to the test deviance (TDEV) the best model was m2, closely followed by m3, and both were better than m1. Hence the transformation and adaptive smoothing methods substantially improve the centiles in terms of both smoothness and goodness of fit.

Further investigation showed that the smaller deviance of the later models relative to model m1 was due to improved prediction of the μ curve—this is unsurprising given its very high curvature in early life (see Figures 1a and 8a). Conversely the power transformation of age in the predictors of σ, ν and τ in model m2 made negligible difference. This is in agreement with the results of [23]. Using different transformations of age for the different distribution parameters might potentially reduce the deviance. However, the adaptive smoothing of age in the predictors of σ and ν in models m3, and m4 considerably reduced the deviance, while for τ there was little or no change. The explanation may be that there is high curvature in the predictor of σ at two age intervals, 0–2 and 13–16 years, and in ν at 7–9 years (see Figure 8b,c). These turning points may in turn provide the opportunity to simplify the curves by making them closer to piecewise linear, which reduces the degrees of freedom needed to define them and hence the corresponding smoothing penalty.

### 5.3 Height in Dutch Boys

This example compares the transformation and adaptive smoothing methods with the Cole transformation as described in Section 3.3. The variables are height (in cm) and age (in years) for 6885 boys from the Fourth Dutch Growth Study, [19, 20] and the data are available in the data frame dbhh in the **gamlss.data** R package.

The distribution chosen was BCTo and models m1 and m2 were as in Section 5.2, but the optimal power transform of age in m2 was the square‐root transformation $t=age^{1/2}$. The fitted predictor of μ from m2 was used as the Cole transformation of age, extracted in R as Ct = m2$mu.lp. Model m3 was obtained by replacing t by Ct in m2. It was found that the σ curve in m2 and m3 was overfitted, so the df for σ was reduced to 6, resulting in smoother fitted curves while maintaining adequate fit as judged by the Q statistics. The optimal value of nseg.sp in the adaptive smoothing model m4 (searching over values from 1 to 5) was nseg.sp=5. The resulting centile plots, plots of the power and Cole transformations, parameter predictors and Q statistics of the residuals are given in Figures C1 to C4 in Supporting Information C. The conclusion is that the models m2, m3, and m4 give very similar results for the predictors and centiles.

## 6 Simulations to Compare Transformations and Adaptive Smoothing

In this section, we use simulation to compare the performance of the transformation and adaptive smoothing methods for improving centile estimation of BMI. For each of the bmi fitted models m2, m3, and m4 in Section 5.2 (each treated as the “true” model), we simulated 1000 samples each of size 7294, using the given values of age and simulating bmi. We then refitted models m1, m2, m3, and m4 from Section 5.2.

In centile estimation, the focus is often on the accuracy and smoothness of the outer centiles, so we investigated the 3rd and 97th centiles. To assess the accuracy of the fitted centiles we calculated the root mean square error (rmse) over the 7294 observations from the centile of each of the four refitted models to the centile of the “true” model. We then found the mean value of rmse from the 1000 simulations, for each combination of refitted model and “true” model. The results are given in Table 3 for the 3rd and 97th centiles. The conclusion is essentially that if the ‘true model’ is m2, m3 or m4 then the corresponding refitted model m2, m3 or m4 provides the most accurate centiles on average.

**TABLE 3:** Mean rmse from the fitted to the “true” model 97% and 3% centiles, from 1000 simulations from each “true” bmi model m2, m3, and m4.

			97%			3%	
			True			True	
		m2	m3	m4	m2	m3	m4
Fitted	m1	0.247	0.257	0.294	0.117	0.129	0.128
	m2	0.195	0.249	0.298	0.081	0.097	0.102
	m3	0.223	0.244	0.266	0.089	0.094	0.098
	m4	0.241	0.254	0.261	0.095	0.100	0.099

We also found that the GAIC information criteria are not suitable for choosing between transformation and adaptive smoothing models, if the objective is accurate 3rd and 97th centiles. This was because for “true” model m2, the refitted model m4 almost always had a lower GAIC(4) despite its centiles fitting much worse than for m2.

To assess smoothness of the centiles we counted the number of turning points and “bumps” of each refitted centile. The results are given in Supporting Information D. The conclusion was that the typical refitted model m2 is smoothest, followed by m3, m4 and then m1.

## 7 Conclusions

Two methods of improving the centile estimation of Y conditional on X are considered. The first method transforms the explanatory variable X to T and fits smooth distribution parameter predictors of Y conditional on T. Transformations (2) are well suited to centile estimation where there is high curvature in a parameter predictor at low values of X (as in the BMI analysis in Section 5.2), while transformations (3) and (4) are well suited to centiles where there is high curvature around an interior value of X, for example a turning point in the μ curve (as in the triceps analysis in Section 5.1). The Cole transformation (Section 3.3) is suited to a μ curve that is monotonic and has high curvature in more than one region of X (see the height example in Section 5.3).

The second method is adaptive smoothing, where the predictor of each distribution parameter of Y is adaptively smoothed against X by allowing the smoothing parameter itself to vary continuously with X. The results show that adaptive smoothing is suited to all forms of high curvature.

In the triceps example in Section 5.1, both transformation and adaptive smoothing produced smooth and well‐fitting centiles that improve on those previously fitted [2]. In the bmi example in Section 5.2, adaptive smoothing identified a local peak in the 97th centile that has not previously been reported. It appears that adaptive smoothing works better than transformation where the parameter predictor has high curvature in two or more regions of X, or where two parameter predictors have high curvature in different regions of X (see e.g., Figure 8). However, the transformation method could be generalized to more flexible transformations and/or different transformations for the different distribution parameters.

However, the adaptively smoothed curves tend to be closer to piecewise linear—and hence less smooth—than the conventionally smoothed curves, and this sharpens the turning points such as those for the σ and ν curves in Figure 8. It is this that underlies the local peak on the 97th centile in Figure 6d. The reason why the adaptively smoothed curves tend towards piecewise linearity is that the smoothing process is flexible enough to allow it, and it has the side effect of reducing the corresponding df and hence the associated smoothing penalty. The bmi results in Table 2 confirm this—$df_{\sigma }$ and $df_{\nu }$ are consistently smaller for models m3, and m4 than for m1 and m2. And the same is true for the triceps results in Table 1—$df_{\mu }$ is appreciably smaller for M3, and M4 than for M1 and M2, and the adaptively smoothed median curves in Figure 4 are linear past age 25. Adaptive smoothing works by making the distribution parameter predictor curves simpler, but not necessarily smoother.

This idea is backed up by the simulations in Section 6, which showed that transformation gave the most accurate 3rd and 97th centiles when the true model was smooth, while adaptive smoothing gave the most accurate centiles when the true model was not smooth. The simulations also showed that for estimating accurate centiles, the GAIC‐based information criteria are unsuitable for choosing between transformation and adaptive smoothing models. This accords with the idea that adaptive smoothing focuses on reducing the df.

The conclusion is that both transformation and adaptive smoothing are useful for improved centile fitting. However, adaptive smoothing tends to “linearise” segments of the underlying predictor curves, and this complicates model comparison. The adaptively smoothed curves tend to be less smooth and potentially overfitting, while the transformation curves are smoother but potentially underfitting. The choice between them can be made by comparing the visual smoothness of their centiles, their deviance values, and their residual diagnostics (i.e., Z and Q statistics and worm plots). Alternatively cross‐validation methods, for example, 10‐fold cross‐validation, could be used.

## Funding

The authors have nothing to report.

## Conflicts of Interest

The authors declare no conflicts of interest.

## Supporting information




**Data S1.** Supporting Information A.

**Data S2.** Supporting Information B.

**Data S3.** Supporting Information C.

**Data S4.** Supporting Information D.

**Data S5.** Supporting Information E.

## Data Availability

The data that support the findings of this study are available in the publicly available R package gamlss.
