# Supplementary material for: Improved Centile Estimation by Transformation And/Or Adaptive Smoothing of the Explanatory Variable
Source: Stat Med. 2026 Feb 5;45(3-5):e70414. doi: 10.1002/sim.70414 (PMC12874224; doi:10.1002/sim.70414)
Supplement: Supplementary file 1 — Data S1. Supporting Information A. [file SIM-45-0-s005.pdf]

## Supplementary Materials A: triceps output and R code

## Figure A1: Z statistics for models M2, M3 and M4

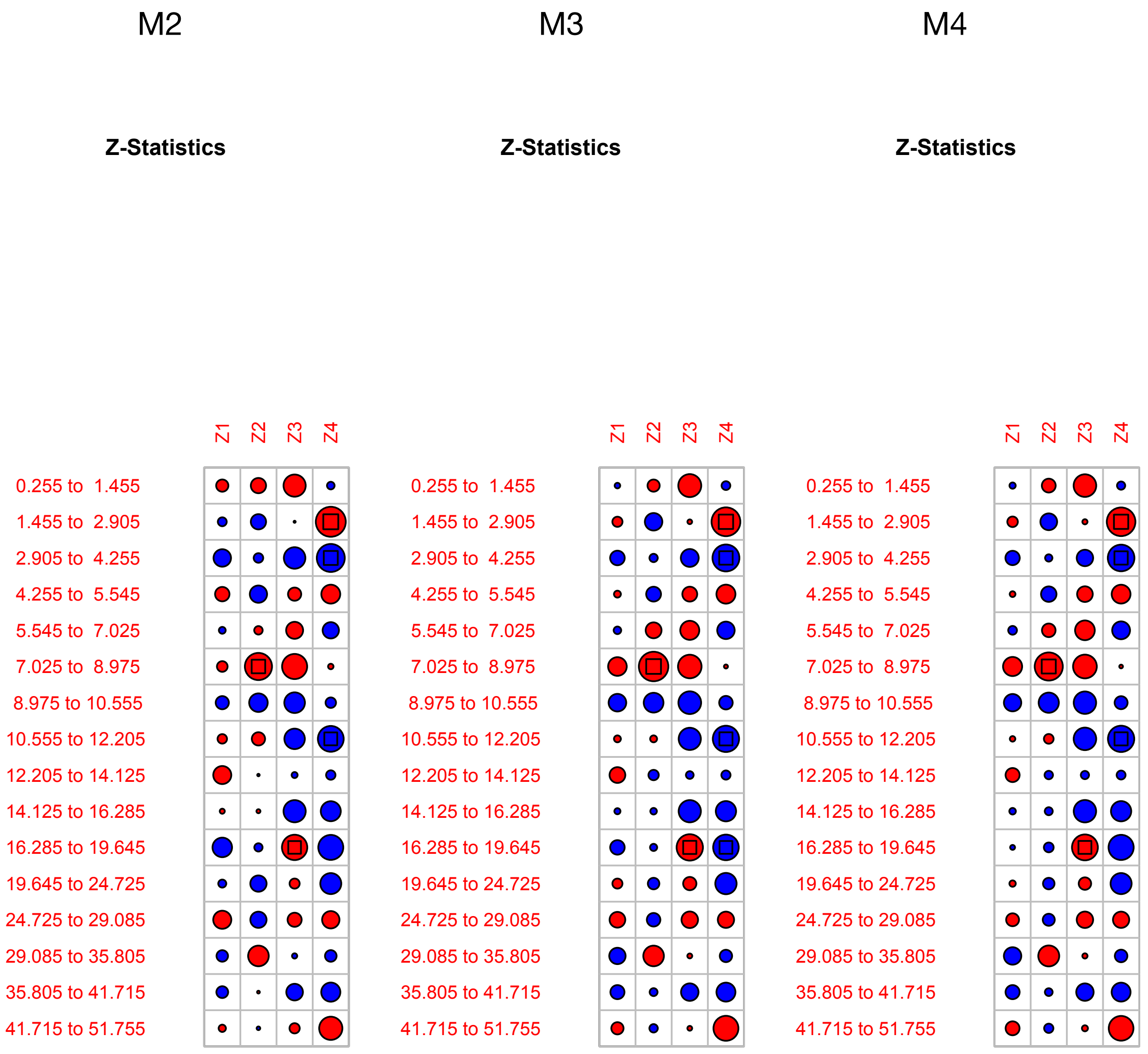

## Q and Z statistics

```
Model M2

> round(Q.stats(M2, xvar=triceps$age, n.inter=16),2)

                         Z1    Z2    Z3    Z4 AgostinoK2   N
 0.255 to  1.455  -0.46 -0.71 -1.50  0.18       2.29  56
 1.455 to  2.905   0.24  0.71  0.01 -2.74       7.53  56
 2.905 to  4.255   0.93  0.28  1.41  2.39       7.73  55
 4.255 to  5.545  -0.63  0.91 -0.53 -1.09       1.47  56
 5.545 to  7.025   0.14 -0.22 -0.88  0.81       1.43  56
 7.025 to  8.975  -0.35 -2.25 -1.92 -0.09       3.71  55
 8.975 to 10.555   0.53  1.05  1.30  0.33       1.80  56
10.555 to 12.205  -0.27 -0.54  1.28  2.03       5.76  56
12.205 to 14.125  -0.99  0.02  0.11  0.27       0.08  57
14.125 to 16.285  -0.08 -0.05  1.50  1.22       3.75  55
16.285 to 19.645   1.17  0.21 -1.96  1.91       7.48  55
19.645 to 24.725   0.20  0.81 -0.31  1.34       1.90  56
24.725 to 29.085  -1.00  0.80 -0.60 -0.92       1.22  56
29.085 to 35.805   0.40 -1.30  0.08  0.41       0.17  55
35.805 to 41.715   0.42 -0.02  0.84  1.12       1.96  57
41.715 to 51.755  -0.16  0.04 -0.32 -1.65       2.81  55
TOTAL Q stats      5.78 11.45 19.73 31.36      51.09 892
df for Q stats     7.14 13.21 12.88 16.00      28.88   0
p-val for Q stats  0.58  0.59  0.10  0.01       0.01   0
```

```
Model M3

> round(Q.stats(M3, xvar=triceps$age, n.inter=16),2)

                         Z1    Z2    Z3    Z4 AgostinoK2   N
 0.255 to  1.455   0.09 -0.45 -1.61  0.23       2.65  56
 1.455 to  2.905  -0.31  0.93 -0.07 -2.53       6.42  56
 2.905 to  4.255   0.65  0.21  0.97  2.22       5.86  55
 4.255 to  5.545  -0.15  0.67 -0.67 -1.10       1.67  56
 5.545 to  7.025   0.17 -0.77 -1.11  0.93       2.08  56
 7.025 to  8.975  -1.06 -2.66 -1.69 -0.05       2.87  55
 8.975 to 10.555   0.95  1.19  1.57  0.56       2.79  56
10.555 to 12.205  -0.15 -0.15  1.50  2.11       6.70  56
12.205 to 14.125  -0.76  0.33  0.18  0.25       0.10  57
14.125 to 16.285   0.11  0.13  1.51  1.26       3.86  55
16.285 to 19.645   0.64  0.16 -2.12  2.00       8.49  55
19.645 to 24.725  -0.31  0.40 -0.54  1.38       2.20  56
24.725 to 29.085  -0.75  0.56 -0.83 -0.81       1.35  56
29.085 to 35.805   0.82 -1.30 -0.07  0.46       0.22  55
35.805 to 41.715   0.60  0.19  0.93  1.10       2.07  57
41.715 to 51.755  -0.45  0.22 -0.07 -1.90       3.62  55
TOTAL Q stats      5.51 13.05 21.49 31.47      52.96 892
df for Q stats     8.52 13.11 13.47 16.00      29.47   0
p-val for Q stats  0.75  0.45  0.08  0.01       0.01   0
```

**Model M4**

```
> round(Q.stats(M4, xvar=triceps$age, n.inter=16),2)

                         Z1    Z2    Z3    Z4 AgostinoK2   N
 0.255 to  1.455   0.12 -0.58 -1.56  0.21       2.48  56
 1.455 to  2.905  -0.34  0.86 -0.08 -2.50       6.25  56
 2.905 to  4.255   0.62  0.16  0.82  2.16       5.36  55
 4.255 to  5.545  -0.10  0.73 -0.75 -1.08       1.73  56
 5.545 to  7.025   0.23 -0.56 -1.19  0.97       2.35  56
 7.025 to  8.975  -1.11 -2.41 -1.72 -0.04       2.97  55
 8.975 to 10.555   0.92  1.25  1.53  0.51       2.61  56
10.555 to 12.205  -0.09 -0.29  1.55  2.11       6.85  56
12.205 to 14.125  -0.60  0.22  0.21  0.24       0.10  57
14.125 to 16.285   0.13  0.19  1.53  1.25       3.91  55
16.285 to 19.645   0.07  0.29 -2.05  1.93       7.91  55
19.645 to 24.725  -0.12  0.40 -0.47  1.35       2.05  56
24.725 to 29.085  -0.51  0.44 -0.82 -0.81       1.33  56
29.085 to 35.805   0.93 -1.34 -0.08  0.48       0.24  55
35.805 to 41.715   0.60  0.18  0.92  1.09       2.04  57
41.715 to 51.755  -0.59  0.26 -0.11 -1.86       3.48  55
TOTAL Q stats      4.91 11.84 21.15 30.52      51.67 892
df for Q stats     8.74 13.10 13.49 16.00      29.49   0
p-val for Q stats  0.83  0.55  0.08  0.02       0.01   0
```

# R code for triceps

```
# This file contains the triceps R code


# First the R code for Figures 3 and 4

# Second the R code for selecting the models


##################################################################
```

# # First the R code for Figures 3 and 4

```
rm(list=ls())


# garbage collection
gc()

library(gamlss)

library(devtools)
devtools::install_github("mstasinopoulos/GAMLSS-Additive-terms-2")
library(gamlss.add2)



triceps<-read.table("~/Documents/BOB_2025_home/Centile_paper_02_02_2025/
Centile_paper_02_12_2024_R_code_REVISED/
NEW_Gambian_triceps_analysis_07_12_2024/Gambia triceps.txt", header=T,
na.strings="NA")



head(triceps)
dim(triceps)





##################################################################
##################################################################
##################################################################
```

```
# Plot Figure 3


# Figure 3, all the 4 centile plots for tricep:

# Figure 3(a) no transformation (model M1)
# Figure 3(b) transformation (model M2)
# Figure 3(c) SOP1  (model M3  with nseg=20 and nseg.sp=5)
# Figure 3(d) SOP2, (model M4  with nseg=20, nseg.sp=8)


op <- par(mfrow=c(2,2))

#################################################################

# Figure 3(a) no transformation (model M1)

M1 <- gamlss(tricep~pb(age),sigma.fo=~pb(age), nu.fo=~pb(age),  family=BCCGo, data=triceps)

centiles(M1, xvar=triceps$age,cent=c(3,10,25,50,75,90,97),legend=FALSE, main="(a) no transform",xlab="age",ylab="tricep", cex=0.4)


#################################################################

# Figure 3(b) transformation (model M2)


age <- triceps$age
t <- 8 + (log (1 + 0.56*abs(age-8)))*sign(age-8)

triceps$t <- t

M2 <- gamlss(tricep~pb(t),sigma.fo=~pb(t), nu.fo=~pb(t), family=BCCGo,, data=triceps, n.cyc=200)

centiles(M2, xvar=triceps$age,cent=c(3,10,25,50,75,90,97),legend=FALSE, main="(b) transform",xlab="age",ylab="tricep", points=FALSE)


#################################################################
```

```
# Figure 3(c)  centiles with SOP, chosen distribution BCCGo
# with nseg=20 and nseg.sp=5

M3 <- gamlss(tricep~SOP(~ ad(age, nseg = 20, nseg.sp = 5)),
sigma.fo=~SOP(~ ad(age, nseg = 20, nseg.sp = 5)),
nu.fo=~SOP(~ ad(age, nseg = 20, nseg.sp = 5)),
family=BCCGo, data=triceps)

centiles(M3, xvar=triceps$age,cent=c(3,10,25,50,75,90,97),legend=FALSE, main="(c)
SOP1",xlab="age",ylab="tricep", points=FALSE)



###################################################################


# Figure 3(d)  centiles with SOP, chosen distribution BCCGo
# with nseg=20 and nseg.sp=8

M4 <- gamlss(tricep~SOP(~ ad(age, nseg = 20, nseg.sp = 8)),
sigma.fo=~SOP(~ ad(age, nseg = 20, nseg.sp = 8)),
nu.fo=~SOP(~ ad(age, nseg = 20, nseg.sp = 8)),
family=BCCGo, data=triceps)

centiles(M4, xvar=triceps$age,cent=c(3,10,25,50,75,90,97),legend=FALSE, main="(c)
SOP2",xlab="age",ylab="tricep", points=FALSE)



###################################################################



par(op)



###################################################################
###################################################################
###################################################################
###################################################################
```

```
# Figure 4

op <- par(mfrow=c(1,2))

# Figure 4(a) transformation from age to t

min(triceps$age)
max(triceps$age)

agenew <- seq(0,52,0.02)

tnew <- 8 + (log (1 + 0.56*abs(agenew-8)))*sign(agenew-8)

plot(tnew ~ agenew, type="l",xlab="age",ylab="t",main="(a) transformation, t")



####################################################################
####################################################################


# Figure 4(b) centiles of tricep against t, using model m2

t <- 8 + (log (1 + 0.56*abs(age-8)))*sign(age-8)

triceps$t <- t

M2 <- gamlss(tricep~pb(t),sigma.fo=~pb(t), nu.fo=~pb(t), family=BCCGo,, data=triceps)

centiles(M2, xvar=triceps$t,cent=c(3,10,25,50,75,90,97),xlab="t",ylab="tricep",main="(b)
centiles of tricep against t", cex=0.4)

par(op)

####################################################################
####################################################################
####################################################################
####################################################################

# Supplementary Figure A1
```

```
op <- par(mfrow=c(1,3))


round(Q.stats(M2, xvar=triceps$age, n.inter=16),2)
round(Q.stats(M3, xvar=triceps$age, n.inter=16),2)
round(Q.stats(M4, xvar=triceps$age, n.inter=16),2)

par(op)



##########################################################################
##########################################################################
##########################################################################
##########################################################################


# Deviance and
# df's for mu, sigma, nu


GAIC(M1,M2,M3,M4,k=0)


M1$mu.df
M2$mu.df
M3$mu.df
M4$mu.df


M1$sigma.df
M2$sigma.df
M3$sigma.df
M4$sigma.df


M1$nu.df
M2$nu.df
M3$nu.df
M4$nu.df



##########################################################################
##########################################################################
##########################################################################
##########################################################################
##########################################################################
##########################################################################
##########################################################################
```

# Second the R code for selecting the models

```
#####################################################################

# Figure 3(a)  centiles (without transformation or adaptive smoothing)

M1 <- gamlss(tricep~pb(age),sigma.fo=~pb(age), nu.fo=~pb(age),  family=BCCGo,,
data=triceps, n.cyc=200)

centiles(M1, xvar=triceps$age,cent=c(3,10,25,50,75,90,97), cex=0.4)

#####################################################################
#####################################################################

# Figure 3(b)  centiles with transformation (with chosen distribution BCCGo)
# (NOTE comparison with BCPEo and BCTo is given at the end of this file)

#####################################################################

# Optim search for best power parameter with BCCGo and GAIC(4)

# Initial guess for the transformation parameter is 0.9

t <- 8 + (log (1 + 0.9*abs(age-8)))*sign(age-8)

triceps$t <- t

k1 <- 4

mbccgA <- gamlss(tricep~pb(t),sigma.fo=~pb(t), nu.fo=~pb(t),   family=BCCGo,
data=triceps, n.cyc=200)
```

```
fnBCCG<- function(p)
      {
triceps$t <- 8 + (log (1 + p[1]*abs(age-8.0)))*sign(age-8.0)


mbccgN <- gamlss(tricep~pb(t),sigma.fo=~pb(t), nu.fo=~pb(t), family=BCCGo,
data=triceps, n.cyc=200, start.from=mbccgA)

      mbccgA <- mbccgN
    cat("p=", p, " and GAIC=", GAIC(mbccgN, k=k1), "\n")
     GAIC(mbccgN, k=k1)
     }




op1 <- optim(par=c(0.9), fnBCCG, method="L-BFGS-B", lower=c(0.3), upper=c(1.5),
control = list(ndeps = 0.002, maxit = 50, factr=1e11))


# NOTE factr=1e11 seems sufficiently small (1e10 not needed)


op1$par
op1$value


##################################################################


# Figure 3(b) centiles (with transformation)

age <- triceps$age
t <- 8 + (log (1 + 0.56*abs(age-8)))*sign(age-8)

triceps$t <- t

M2 <- gamlss(tricep~pb(t),sigma.fo=~pb(t), nu.fo=~pb(t), family=BCCGo,, data=triceps,
n.cyc=200)

centiles(M2, xvar=triceps$age,cent=c(3,10,25,50,75,90,97),legend=FALSE, main="(b)
transform",xlab="age",ylab="tricep", points=FALSE)


##################################################################
##################################################################
##################################################################
##################################################################


# Figures 3(c) and 3(d)  centiles with SOP, chosen distribution BCCGo
```

```
####################################################################
####################################################################


# Figure 3(c) SOP1
# Figure 3(d) SOP2


####################################################################
####################################################################


# Figures 3(c) and 3(d)  (SOP1 and SOP2)

# The function: find_knot() selects the value of nseg.sp
# which gives the lowest value of GAIC
# Function find_knot() requires the function model() to specify the model




########################################################################
########
model <- function(nsp = 1, data=triceps)
  {
    M<- gamlss(tricep~SOP(~ ad(age, nseg=20, nseg.sp = nsp)),
               sigma.fo=~SOP(~ ad(age, nseg=20, nseg.sp = nsp)),
               nu.fo=~SOP(~ ad(age, nseg=20, nseg.sp = nsp)),
               family=BCCGo, data=triceps, trace=F, n.cyc=100)
    M
  }
########################################################################
########
find_knot <- function(from=1, to=10, k=4)
  {
    if(is.null(model)) stop("the model is required")
    models <-  list()
    for (i in from:to)
    {
    assign("nsp", i, envir = .GlobalEnv)
    models[[i]] <- m0 <-  model(i)
    assign("m0",  models[[i]], envir = .GlobalEnv)
    }
    GAICs <- sapply(models, GAIC, k=k)
    GAICs0 <- sapply(models, GAIC, k=0)
    GAICs2 <- sapply(models, GAIC, k=2)
    GAICs4 <- sapply(models, GAIC, k=4)
    GAICsB <- sapply(models, GAIC, k=log(length(triceps$age)))
    pos <- which.min(GAICs)
    out <- list(model=models[[pos]], GAIC=GAICs, min=pos, GAIC0=GAICs0,
GAIC2=GAICs, GAIC4=GAICs4, GAICB=GAICsB)
```

```
    out
}

#####################################################################
#####################################################################

# Fitting the 3rd and 4th models
#####################################################################

M <- find_knot(from=1, to=10, k=4)
M
#####################################################################

#  model M4
nsp <<- nsp <- which.min(M$GAIC4)

M4 <- gamlss(tricep ~ SOP(~ ad(age, nseg=20, nseg.sp = nsp)),
             sigma.fo=~SOP(~ ad(age, nseg=20, nseg.sp = nsp)),
             nu.fo=~SOP(~ ad(age, nseg=20, nseg.sp = nsp)),
             family=BCCGo, data=triceps, n.cyc=100, trace=FALSE)

centiles(M4, xvar=triceps$age,cent=c(3,10,25,50,75,90,97),legend=FALSE, main="(c)
SOP2",xlab="age",ylab="tricep", points=FALSE)


#####################################################################

# model M3
nsp2 <<- nsp2<- which.min(M$GAIC4[1:5])

M3 <- gamlss(tricep ~ SOP(~ ad(age, nseg=20, nseg.sp = nsp2)),
             sigma.fo=~SOP(~ ad(age, nseg=20, nseg.sp = nsp2)),
             nu.fo=~SOP(~ ad(age, nseg=20, nseg.sp = nsp2)),
             family=BCCGo, data=triceps, n.cyc=100, trace=FALSE)


centiles(M3, xvar=triceps$age,cent=c(3,10,25,50,75,90,97),legend=FALSE, main="(c)
SOP1",xlab="age",ylab="tricep", points=FALSE)


#####################################################################
#####################################################################
#####################################################################
#####################################################################



# Supplementary Figure A1



op <- par(mfrow=c(1,3))
```

```
round(Q.stats(M2, xvar=triceps$age,n.inter=16),2)
round(Q.stats(M3, xvar=triceps$age,n.inter=16),2)
round(Q.stats(M4, xvar=triceps$age,n.inter=16),2)


par(op)



###################################################################
###################################################################
###################################################################
###################################################################
###################################################################
###################################################################
###################################################################
###################################################################



# Optim search for best transformation with BCTo and GAIC(4)

# Initial guess for the transformation parameter is 0.5

age <- triceps$age
t <- 8 + (log (1 + 0.5*abs(age-8)))*sign(age-8)


triceps$t <- t



k1 <- 4


mbctA <- gamlss(tricep~pb(t),sigma.fo=~pb(t), nu.fo=~pb(t),  tau.fo=~pb(t),  family=BCTo,
data=triceps, n.cyc=200)


fnBCT<- function(p)
     {triceps$t <- 8 + (log (1 + p[1]*abs(age-8.0)))*sign(age-8.0)

mbctN <- gamlss(tricep~pb(t),sigma.fo=~pb(t), nu.fo=~pb(t),  tau.fo=~pb(t), family=BCTo,
data=triceps, n.cyc=200, start.from=mbctA)

     mbctA <- mbctN
   cat("p=", p, " and GAIC=", GAIC(mbctN, k=k1), "\n")
    GAIC(mbctN, k=k1)
    }
```

```
op2 <- optim(par=c(0.5), fnBCT, method="L-BFGS-B", lower=c(0.3), upper=c(0.9), control
= list(ndeps = 0.002, maxit = 50, factr=1e12))


# NOTE factr=1e12 seems sufficiently small (1e11 or 1e10 not needed)


op2$par
op2$value


#####################################################################


# Optim search for best transformation with BCPEo and GAIC(4)

# Initial guess for the transformation parameter is 0.9


t <- 8 + (log (1 + 0.9*abs(age-8)))*sign(age-8)


triceps$t <- t


k1 <- 4


mbcpeA <- gamlss(tricep~pb(t),sigma.fo=~pb(t), nu.fo=~pb(t),  tau.fo=~pb(t),
family=BCPEo, data=triceps, n.cyc=200)


fnBCPE<- function(p)
      {triceps$t <- 8 + (log (1 + p[1]*abs(age-8.0)))*sign(age-8.0)

mbcpeN <- gamlss(tricep~pb(t),sigma.fo=~pb(t), nu.fo=~pb(t),  tau.fo=~pb(t),
family=BCPEo, data=triceps, n.cyc=200, start.from=mbcpeA)

      mbcpeA <- mbcpeN
    cat("p=", p, " and GAIC=", GAIC(mbcpeN, k=k1), "\n")
     GAIC(mbcpeN, k=k1)
     }
```

```
op3 <- optim(par=c(0.9), fnBCPE, method="L-BFGS-B", lower=c(0.3), upper=c(1.5),
control = list(ndeps = 0.002, maxit = 50, factr=1e12))


# NOTE factr=1e12 seems sufficiently small (1e11 or 1e10 not needed)


op3$par
op3$value



########################################################################



# Comparing the power parameter and GAIC values for transformation model
# with the distributions BCCGo, BCTo and BCPEo

op1$par
op1$value

op2$par
op2$value

op3$par
op3$value
```
