# Supplementary material for: Improved Centile Estimation by Transformation And/Or Adaptive Smoothing of the Explanatory Variable
Source: Stat Med. 2026 Feb 5;45(3-5):e70414. doi: 10.1002/sim.70414 (PMC12874224; doi:10.1002/sim.70414)
Supplement: Supplementary file 2 — Data S2. Supporting Information B. [file SIM-45-0-s002.pdf]

Supplementary Materials B: BMI output and R code

Figure B1: 95% confidence intervals for the 2.5%, 50% and 97.5% centiles for models m1, m2, m3 and m4 (obtained from 1000 bootstrap samples).

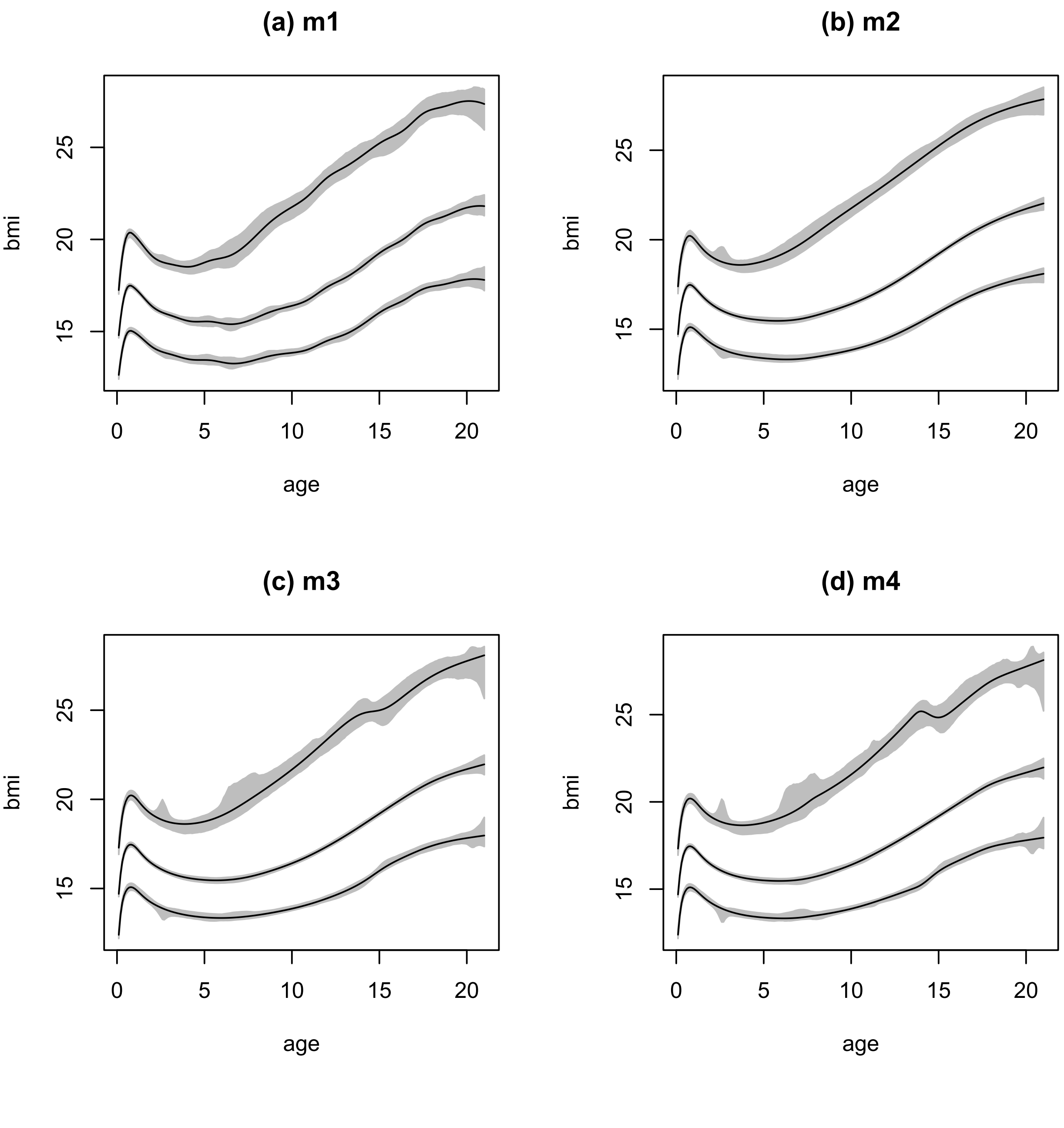

[Note that in 3 and 17 of the 1000 bootstrap samples the fitted m3 and m4 models, respectively, failed to converge and were replaced to obtain 1000 fitted models. Consequently the confidence intervals, especially for m4, may be wider than shown above.]

Figure B2: Z statistics for models m2, m3 and m4

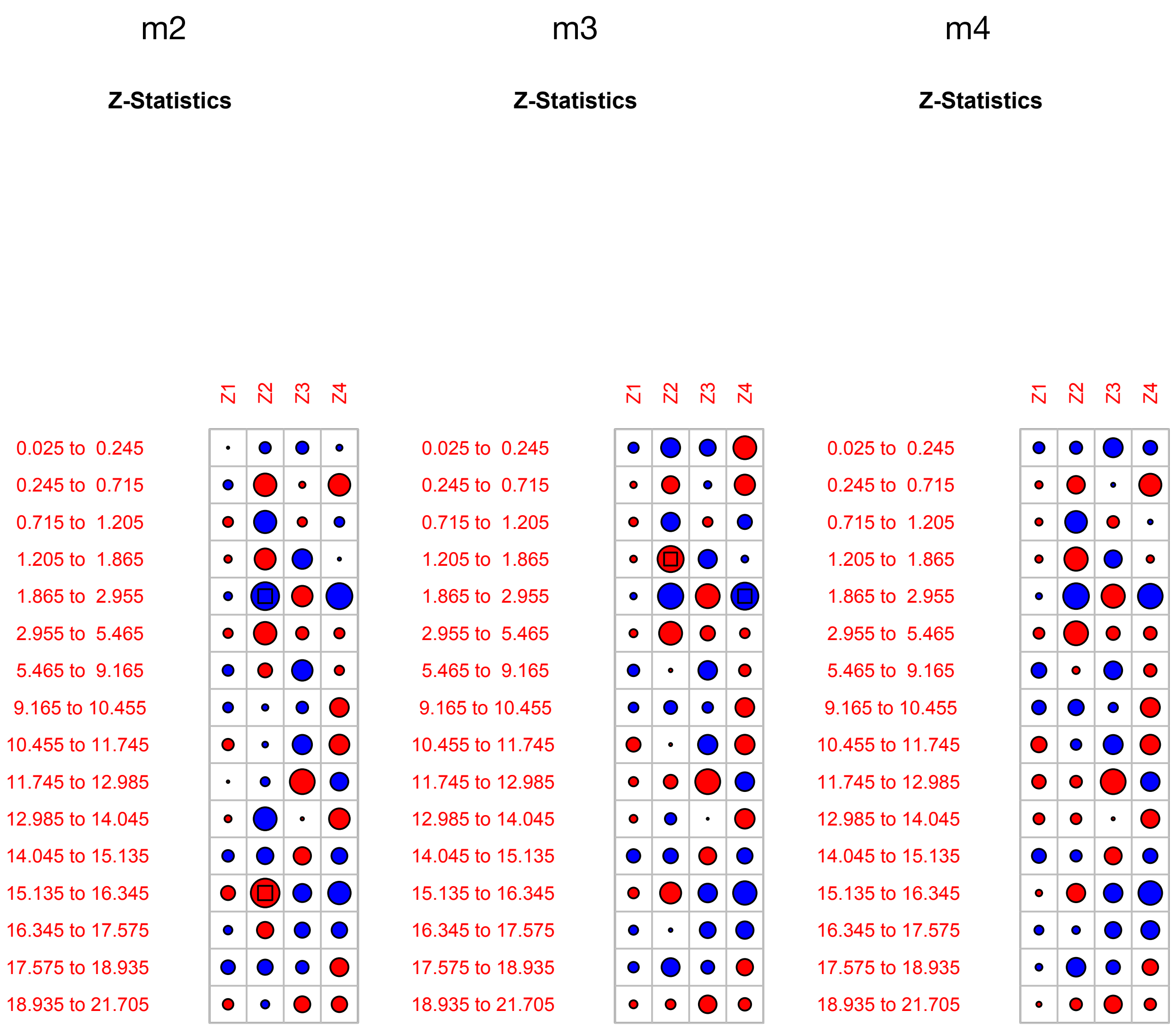

Q and Z statistics

```
Model m2

> round(Q.stats(m2, xvar=dbbmi$age,n.inter=16),2)

                          Z1    Z2    Z3    Z4 AgostinoK2    N
 0.025 to  0.245  -0.02  0.38  0.44  0.11       0.20  473
 0.245 to  0.715   0.25 -1.48 -0.12 -1.38       1.92  442
 0.715 to  1.205  -0.29  1.45 -0.26  0.28       0.15  458
 1.205 to  1.865  -0.16 -1.29  1.12  0.03       1.25  451
 1.865 to  2.955   0.19  2.25 -1.24  1.96       5.36  455
 2.955 to  5.465  -0.26 -1.48 -0.46 -0.31       0.30  460
 5.465 to  9.165   0.35 -0.57  1.23 -0.26       1.58  455
 9.165 to 10.455   0.29  0.12  0.40 -1.03       1.21  454
10.455 to 11.745  -0.38  0.10  1.09 -1.15       2.52  457
11.745 to 12.985  -0.02  0.24 -1.80  0.92       4.08  455
12.985 to 14.045  -0.14  1.53 -0.03 -1.25       1.55  458
14.045 to 15.135   0.39  0.80 -0.88  0.74       1.32  456
15.135 to 16.345  -0.58 -2.39  0.95  1.47       3.08  455
16.345 to 17.575   0.22 -0.79  0.68  0.70       0.95  455
17.575 to 18.935   0.55  0.67  0.47 -0.95       1.13  457
18.935 to 21.705  -0.33  0.20 -0.75 -0.70       1.06  453
TOTAL Q stats      1.60 23.57 12.28 15.39      27.66 7294
df for Q stats     3.36 12.36 10.46 14.00      24.46    0
p-val for Q stats  0.72  0.03  0.30  0.35       0.30    0

Model m3

> round(Q.stats(m3, xvar=dbbmi$age,n.inter=16),2)

                          Z1    Z2    Z3    Z4 AgostinoK2    N
 0.025 to  0.245   0.31  1.06  0.75 -1.53       2.92  473
 0.245 to  0.715  -0.12 -0.87  0.16 -1.21       1.50  442
 0.715 to  1.205  -0.22  0.99 -0.27  0.59       0.42  458
 1.205 to  1.865  -0.13 -1.98  0.98  0.14       0.98  451
 1.865 to  2.955   0.13  1.87 -1.67  2.13       7.30  455
 2.955 to  5.465  -0.19 -1.54 -0.62 -0.28       0.46  460
 5.465 to  9.165   0.39 -0.04  1.02 -0.40       1.19  455
 9.165 to 10.455   0.28  0.50  0.33 -1.03       1.17  454
10.455 to 11.745  -0.58 -0.03  1.10 -1.11       2.44  457
11.745 to 12.985  -0.25 -0.55 -1.80  1.02       4.28  455
12.985 to 14.045  -0.18  0.38 -0.01 -1.11       1.24  458
14.045 to 15.135   0.53  0.63 -0.85  0.69       1.20  456
15.135 to 16.345  -0.34 -1.30  1.01  1.60       3.58  455
16.345 to 17.575   0.25  0.04  0.74  0.88       1.32  455
17.575 to 18.935   0.32  0.95  0.50 -0.79       0.86  457
18.935 to 21.705  -0.19 -0.29 -0.91 -0.46       1.04  453
TOTAL Q stats      1.50 16.40 13.75 18.16      31.91 7294
df for Q stats     3.29 12.51 12.53 13.09      25.62    0
p-val for Q stats  0.73  0.20  0.36  0.16       0.18    0
```

Model m4

```
> round(Q.stats(m4, xvar=dbbmi$age,n.inter=16),2)

                        Z1    Z2    Z3    Z4 AgostinoK2    N
 0.025 to  0.245   0.36  0.44  1.06  0.55       1.43  473
 0.245 to  0.715  -0.16 -0.91  0.05 -1.43       2.05  442
 0.715 to  1.205  -0.15  1.38 -0.41  0.07       0.17  458
 1.205 to  1.865  -0.16 -1.59  0.88 -0.16       0.80  451
 1.865 to  2.955   0.11  1.92 -1.58  1.77       5.64  455
 2.955 to  5.465  -0.35 -1.67 -0.52 -0.48       0.50  460
 5.465 to  9.165   0.65 -0.16  0.94 -0.46       1.09  455
 9.165 to 10.455   0.55  0.69  0.26 -1.07       1.21  454
10.455 to 11.745  -0.70  0.33  1.07 -1.13       2.41  457
11.745 to 12.985  -0.54 -0.41 -1.80  1.01       4.27  455
12.985 to 14.045  -0.35 -0.34 -0.03 -0.96       0.93  458
14.045 to 15.135   0.58  0.38 -0.85  0.60       1.09  456
15.135 to 16.345  -0.12 -0.99  1.02  1.63       3.69  455
16.345 to 17.575   0.24  0.18  0.77  0.95       1.50  455
17.575 to 18.935   0.14  1.02  0.54 -0.71       0.80  457
18.935 to 21.705  -0.08 -0.41 -0.89 -0.39       0.94  453
TOTAL Q stats      2.39 15.17 13.59 14.91      28.51 7294
df for Q stats     3.89 12.50 12.99 14.00      26.99    0
p-val for Q stats  0.65  0.26  0.40  0.38       0.38    0
```

Note that model m2 has a marginally significant Q statistic (p=0.03) with a highly significant Z2 value for age range 15.1 to 16.3, showing that the variance of the residuals in model m2 was significantly low in age range 15.1 to 16.3, indicating that its fitted σ was too high in that age range.

R code for BMI

```
# This file contains the BMI R code

# First the R code for Figures 5, 6 and 7

# Second the R code for selecting the models

##########################################################################
```

# First the R code for Figures 5, 6 and 7

```
rm(list=ls())

# garbage collection
gc()

library(gamlss)

library(devtools)
devtools::install_github("mstasinopoulos/GAMLSS-Additive-terms-2")
library(gamlss.add2)

data(dbbmi)
head(dbbmi)
dim(dbbmi)

##########################################################################
##########################################################################
##########################################################################
##########################################################################

# First the R code for Figures 5, 6 and 7

##########################################################################

# Figure 5
```

```
# Figure 5, all the 4 centile plots for bmi:
# Figure 5(a) no transformation (model m1)
# Figure 5(b) transformation (model m2)
# Figure 5(c) SOP1 (moded m3 with nseg=50 and nseg.sp=4)
# Figure 5(d) SOP2 (model m4 with nseg=50 and nseg.sp=8)


op <- par(mfrow=c(2,2))


##################################################################



# Figure 5(a) no transformation (model m1)

m1<-
gamlss(bmi~pb(age),sigma.fo=~pb(age),nu.fo=~pb(age),tau.fo=~pb(age),family=BCTo,dat
a=dbbmi)

centiles(m1, xvar=dbbmi$age,cent=c(3,10,25,50,75,90,97), legend=FALSE, main="(a) no
transform",xlab="age",ylab="bmi", cex=0.4)



##################################################################


# Figure 5(b) transformation (model m2)


# Chosen transformation model for bmi

t <- dbbmi$age^(1/3)

dbbmi$t <- t

m2 <- gamlss(bmi~pb(t),sigma.fo=~pb(t), nu.fo=~pb(t), tau.fo=~pb(t),  family=BCTo,
data=dbbmi)

centiles(m2, xvar=dbbmi$age,cent=c(3,10,25,50,75,90,97), legend=FALSE, main="(b)
transform",xlab="age",ylab="bmi", points=FALSE)



##################################################################


# Figure 5(c) SOP1 (model m3)
```

```
# The SOP model with the lowest GAIC(4) for nseg.sp from 1 to 5 and nseg=50
# nseg=50 and nseg.sp=4


ns <- 50
nsp <- 4


m3  <- gamlss(bmi~SOP(~ ad(age, nseg = ns, nseg.sp = nsp)),
sigma.fo=~SOP(~ ad(age, nseg = ns, nseg.sp = nsp)),
nu.fo=~SOP(~ ad(age, nseg = ns, nseg.sp = nsp)),
tau.fo=~SOP(~ ad(age, nseg = ns, nseg.sp = nsp)),
family=BCTo, data=dbbmi)


centiles(m3, xvar=dbbmi$age,cent=c(3,10,25,50,75,90,97), legend=FALSE, main="(c)
SOP1",xlab="age",ylab="bmi", points=FALSE)



#######################################################################


# Figure 5(d) SOP1 (model m4)


# SOP2 model with the lowest GAIC(4) for nseg.sp from 1 to 10 and nseg=50
# nseg=50 and nseg.sp=8


ns <- 50
nsp <- 8


m4 <- gamlss(bmi~SOP(~ ad(age, nseg = ns, nseg.sp = nsp)),
sigma.fo=~SOP(~ ad(age, nseg = ns, nseg.sp = nsp)),
nu.fo=~SOP(~ ad(age, nseg = ns, nseg.sp = nsp)),
tau.fo=~SOP(~ ad(age, nseg = ns, nseg.sp = nsp)),
family=BCTo, data=dbbmi)


centiles(m4, xvar=dbbmi$age,cent=c(3,10,25,50,75,90,97), legend=FALSE, main="(d)
SOP2",xlab="age",ylab="bmi",, points=FALSE)


par(op)



#######################################################################
#######################################################################
#######################################################################
```

```
######################################################################

# Figure 6

op <- par(mfrow=c(1,2))

######################################################################

# Figure 6(a) Transformation from age to t

agenew <- seq(0,22,0.01)
tnew <- agenew^(1/3)

plot(tnew ~ agenew, type="l",xlab="age",ylab="t",main="(a) transformation, t")

######################################################################

# Figure 6(b)

t <- dbbmi$age^(1/3)

dbbmi$t <- t

m2 <- gamlss(bmi~pb(t),sigma.fo=~pb(t), nu.fo=~pb(t), tau.fo=~pb(t),  family=BCTo,
data=dbbmi)

centiles(m2, xvar=dbbmi$t,cent=c(3,10,25,50,75,90,97),xlab="t",ylab="bmi",main="(b)
centiles of bmi against t", cex=0.4)

######################################################################

par(op)

######################################################################
######################################################################
######################################################################
######################################################################
```

Figure 7: fitted parameter predictors against age for models m1, m2, m3, and m4

```
op <- par(mfrow=c(2,2))
plot(dbbmi$age,m4$mu.lp,type="l",main="(a)",ylab="mu",xlab="age",ylim=c(2.59,3.1))
lines(dbbmi$age,m1$mu.lp,col="green")
lines(dbbmi$age,m2$mu.lp,col="red")
lines(dbbmi$age,m3$mu.lp,col="blue")
plot(dbbmi$age,m4$sigma.lp,type="l",main="(b)",ylab="log(sigma)",xlab="age",ylim=c(-2.
7,-2.1))
lines(dbbmi$age,m1$sigma.lp,col="green")
lines(dbbmi$age,m2$sigma.lp,col="red")
lines(dbbmi$age,m3$sigma.lp,col="blue")
plot(dbbmi$age,m4$nu.lp,type="l",main="(c)",ylab="nu",xlab="age",ylim=c(-2.8,0.5))
lines(dbbmi$age,m1$nu.lp,col="green")
lines(dbbmi$age,m2$nu.lp,col="red")
lines(dbbmi$age,m3$nu.lp,col="blue")
plot(dbbmi$age,m4$tau.lp,type="l",main="(d)",ylab="log(tau)",xlab="age",ylim=c(1,4.5))
lines(dbbmi$age,m1$tau.lp,col="green")
lines(dbbmi$age,m2$tau.lp,col="red")
lines(dbbmi$age,m3$tau.lp,col="blue")
par(op)
```

```
####################################################################
####################################################################
####################################################################
####################################################################
```

Figure B2: Q statistics plots for m2, m3, and m4

```
op <- par(mfrow=c(1,3))

round(Q.stats(m2, xvar=dbbmi$age,n.inter=16),2)
round(Q.stats(m3, xvar=dbbmi$age,n.inter=16),2)
round(Q.stats(m4, xvar=dbbmi$age,n.inter=16),2)

par(op)
```

```
#################################################################
#################################################################
#################################################################
#################################################################


# Deviance and
# df's for mu, sigma, nu and tau


GAIC(m1,m2,m3,m4,k=0)


m1$mu.df
m2$mu.df
m3$mu.df
m4$mu.df


m1$sigma.df
m2$sigma.df
m3$sigma.df
m4$sigma.df


m1$nu.df
m2$nu.df
m3$nu.df
m4$nu.df


m1$tau.df
m2$tau.df
m3$tau.df
m4$tau.df


#################################################################
#################################################################
#################################################################
#################################################################
#################################################################
#################################################################
#################################################################
#################################################################
```

Second the R code for selecting the models

```
######################################################################


# Figure 5(a) centiles (without transformation or adaptive smoothing)


m1<-
gamlss(bmi~pb(age),sigma.fo=~pb(age),nu.fo=~pb(age),tau.fo=~pb(age),family=BCTo,dat
a=dbbmi)

centiles(m1, xvar=dbbmi$age,cent=c(3,10,25,50,75,90,97), cex=0.4)



######################################################################
######################################################################


# Figure 5(b)  centiles with transformation (with chosen distribution BCTo)
# (NOTE comparison with BCCGo and BCPEo is given at the end of this file)


######################################################################

# Optim search for best power parameter with BCTo and GAIC(4)

t <- dbbmi$age^0.5

dbbmi$t <- t

k1 <- 4


mbctA <- gamlss(bmi~pb(t),sigma.fo=~pb(t), nu.fo=~pb(t), tau.fo=~pb(t),  family=BCTo,
data=dbbmi, n.cyc=200)


fnBCT<- function(p)
    {dbbmi$t <- dbbmi$age^p[1]


mbctN <- gamlss(bmi~pb(t),sigma.fo=~pb(t), nu.fo=~pb(t), tau.fo=~pb(t),  family=BCTo,
data=dbbmi, n.cyc=200, start.from=mbctA)
```

```
    mbctA <- mbctN
   cat("p=", p, " and GAIC=", GAIC(mbctN, k=k1), "\n")
    GAIC(mbctN, k=4)
    }



op1 <- optim(par=c(.5), fnBCT, method="L-BFGS-B", lower=c(.2), upper=c(1.1),
control = list(maxit = 50, factr=1e10))


op1$par
op1$value



################################################################
################################################################


# Chosen distribution and transformation model for bmi

t <- dbbmi$age^(1/3)

dbbmi$t <- t

m2 <- gamlss(bmi~pb(t),sigma.fo=~pb(t), nu.fo=~pb(t), tau.fo=~pb(t),  family=BCTo,
data=dbbmi, n.cyc=200)

centiles(m2, xvar=dbbmi$age,cent=c(3,10,25,50,75,90,97), points=FALSE)

GAIC(m2,k=4)



################################################################
################################################################
################################################################
################################################################


# Figure 5(c) SOP1
# Figure 5(d) SOP2


################################################################
################################################################
################################################################
################################################################
```

```
# Figures 5(c) and 5(d)  (SOP1 and SOP2)

# The function: find_knot() selects the value of nseg.sp
# which gives the lowest value of GAIC
# Function find_knot() requires the function model() to specify the model


#####################################################################
model <- function(nsp = 1, data=dbbmi)
  {
    M<- gamlss(bmi~SOP(~ ad(age, nseg=50, nseg.sp = nsp)),
              sigma.fo=~SOP(~ ad(age, nseg=50, nseg.sp = nsp)),
              nu.fo=~SOP(~ ad(age, nseg=50, nseg.sp = nsp)),
              tau.fo=~SOP(~ ad(age, nseg=50, nseg.sp = nsp)),
              family=BCTo, data=dbbmi, trace=F, n.cyc=100)
    M
  }
#####################################################################
find_knot <- function(from=1, to=10, k=4)
  {
    if(is.null(model)) stop("the model is required")
    models <-  list()
    for (i in from:to)
    {
    assign("nsp", i, envir = .GlobalEnv)
    models[[i]] <- m0 <-  model(i)
    assign("m0",  models[[i]], envir = .GlobalEnv)
    }
    GAICs <- sapply(models, GAIC, k=k)
    GAICs0 <- sapply(models, GAIC, k=0)
    GAICs2 <- sapply(models, GAIC, k=2)
    GAICs4 <- sapply(models, GAIC, k=4)
    GAICsB <- sapply(models, GAIC, k=log(length(dbbmi$age)))
    pos <- which.min(GAICs)
    out <- list(model=models[[pos]], GAIC=GAICs, min=pos, GAIC0=GAICs0,
GAIC2=GAICs, GAIC4=GAICs4, GAICB=GAICsB)
    out
}
#####################################################################
#####################################################################
#####################################################################

# Selecting the best SOP model with nseg=50

#####################################################################
#####################################################################
Fitting the 3rd and 4th models
#####################################################################
M <- find_knot(from=1, to=10, k=4)
M
```

```
#######################################################################
model m4
nsp <<- nsp <- which.min(M$GAIC4)

m4  <- gamlss(bmi~SOP(~ ad(age, nseg = 50, nseg.sp = nsp)),
sigma.fo=~SOP(~ ad(age, nseg = 50, nseg.sp = nsp)),
nu.fo=~SOP(~ ad(age, nseg = 50, nseg.sp = nsp)),
tau.fo=~SOP(~ ad(age, nseg = 50, nseg.sp = nsp)),
family=BCTo, data=dbbmi, n.cyc=100, trace=FALSE)

centiles(m4, xvar=dbbmi$age,cent=c(3,10,25,50,75,90,97), legend=FALSE, main="(c)
SOP2",xlab="age",ylab="bmi", points=FALSE)

#######################################################################
# model m3
nsp2 <<- nsp2<- which.min(M$GAIC4[1:5])

m3  <- gamlss(bmi~SOP(~ ad(age, nseg = 50, nseg.sp = nsp2)),
sigma.fo=~SOP(~ ad(age, nseg = 50, nseg.sp = nsp2)),
nu.fo=~SOP(~ ad(age, nseg = 50, nseg.sp = nsp2)),
tau.fo=~SOP(~ ad(age, nseg = 50, nseg.sp = nsp2)),
family=BCTo, data=dbbmi, n.cyc=100, trace=FALSE)


centiles(m3, xvar=dbbmi$age,cent=c(3,10,25,50,75,90,97), legend=FALSE, main="(c)
SOP1",xlab="age",ylab="bmi", points=FALSE)



#######################################################################
#######################################################################
#######################################################################




# Optim search for best power parameter with BCPEo and GAIC(4)

t <- dbbmi$age^0.5

dbbmi$t <- t

k1 <- 4


mbcpeA <- gamlss(bmi~pb(t),sigma.fo=~pb(t), nu.fo=~pb(t), tau.fo=~pb(t),
family=BCPEo, data=dbbmi, n.cyc=200)


fnBCPE<- function(p)
     {dbbmi$t <- dbbmi$age^p[1]
```

```
mbcpeN <- gamlss(bmi~pb(t),sigma.fo=~pb(t), nu.fo=~pb(t), tau.fo=~pb(t),
family=BCPEo, data=dbbmi, n.cyc=200, start.from=mbcpeA)

     mbcpeA <- mbcpeN
   cat("p=", p, " and GAIC=", GAIC(mbcpeN, k=k1), "\n")
    GAIC(mbcpeN, k=4)
    }



op2 <- optim(par=c(.5), fnBCPE, method="L-BFGS-B", lower=c(.2), upper=c(1.1),
control = list(maxit = 50, factr=1e10))


op2$par
op2$value


######################################################################



# Optim search for best power parameter with BCCGo and GAIC(4)

t <- dbbmi$age^0.5

dbbmi$t <- t

k1 <- 4


mbccgA <- gamlss(bmi~pb(t),sigma.fo=~pb(t), nu.fo=~pb(t), family=BCCGo, data=dbbmi,
n.cyc=200)


fnBCCG<- function(p)
     {dbbmi$t <- dbbmi$age^p[1]


mbccgN <- gamlss(bmi~pb(t),sigma.fo=~pb(t), nu.fo=~pb(t), family=BCCGo, data=dbbmi,
n.cyc=200, start.from=mbccgA)

     mbccgA <- mbccgN
   cat("p=", p, " and GAIC=", GAIC(mbccgN, k=k1), "\n")
    GAIC(mbccgN, k=4)
    }



op3 <- optim(par=c(.5), fnBCCG, method="L-BFGS-B", lower=c(.2), upper=c(1.1),
control = list(maxit = 50, factr=1e10))
```

```
op3$par
op3$value



###########################################################################


# Comparing the power parameter and GAIC values for transformation model
# with the distributions BCTo, BCPEo and BCCGo

op1$par
op1$value

op2$par
op2$value

op3$par
op3$value
```
