# Supplementary material for: Improved Centile Estimation by Transformation And/Or Adaptive Smoothing of the Explanatory Variable
Source: Stat Med. 2026 Feb 5;45(3-5):e70414. doi: 10.1002/sim.70414 (PMC12874224; doi:10.1002/sim.70414)
Supplement: Supplementary file 3 — Data S3. Supporting Information C. [file SIM-45-0-s003.pdf]

Supplementary Materials C: height output and R code

Figure C1: Centile plots from fitted models (a) m1: no transform, (b) m2: power transform, (c) m3: Cole transform, and (d) SOP.

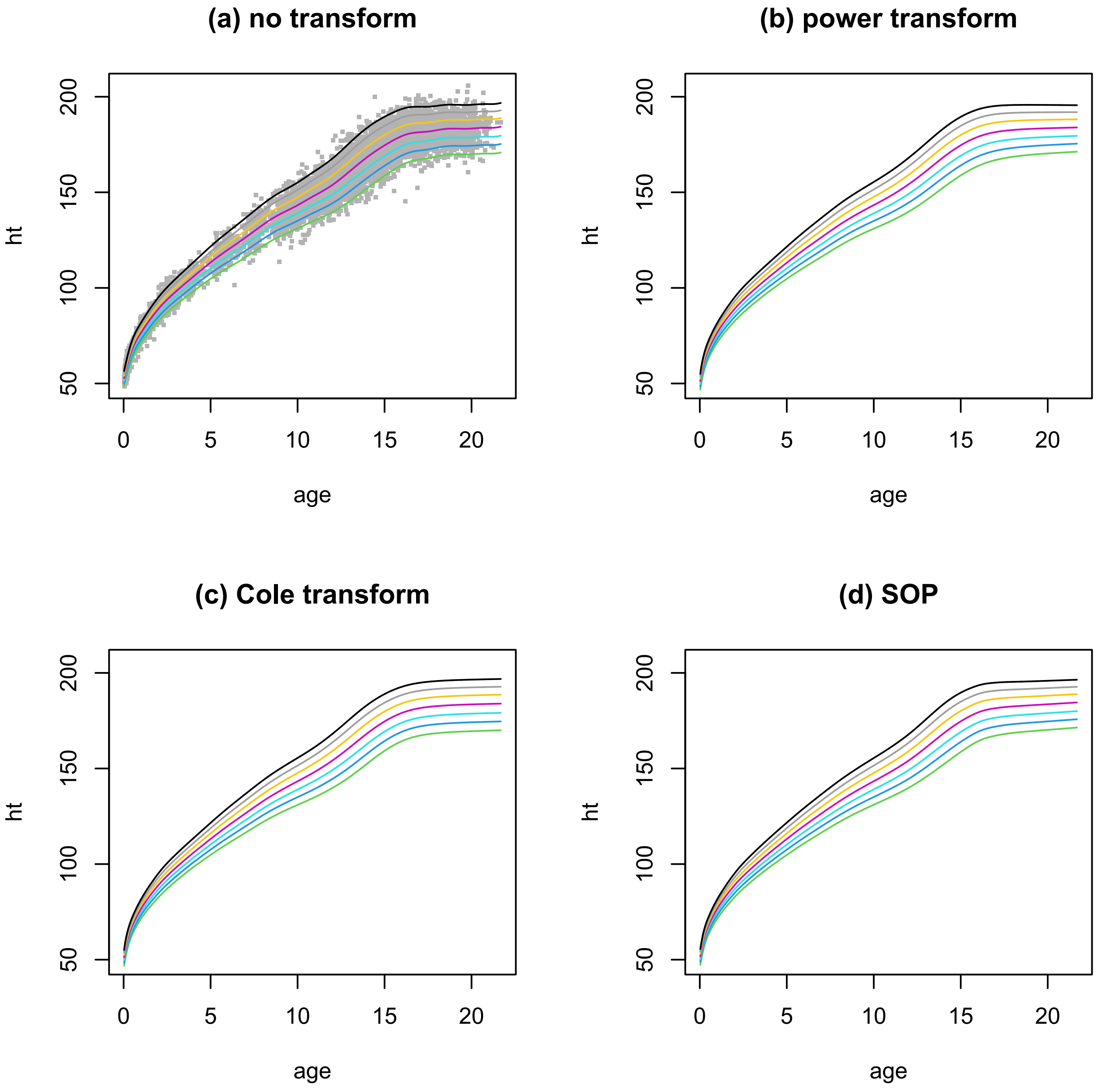

Figure C2: (a) power transformation t and (b) resulting centiles of ht against t, and (c) Cole transformation t and (d) resulting centiles of ht agents Cole t.

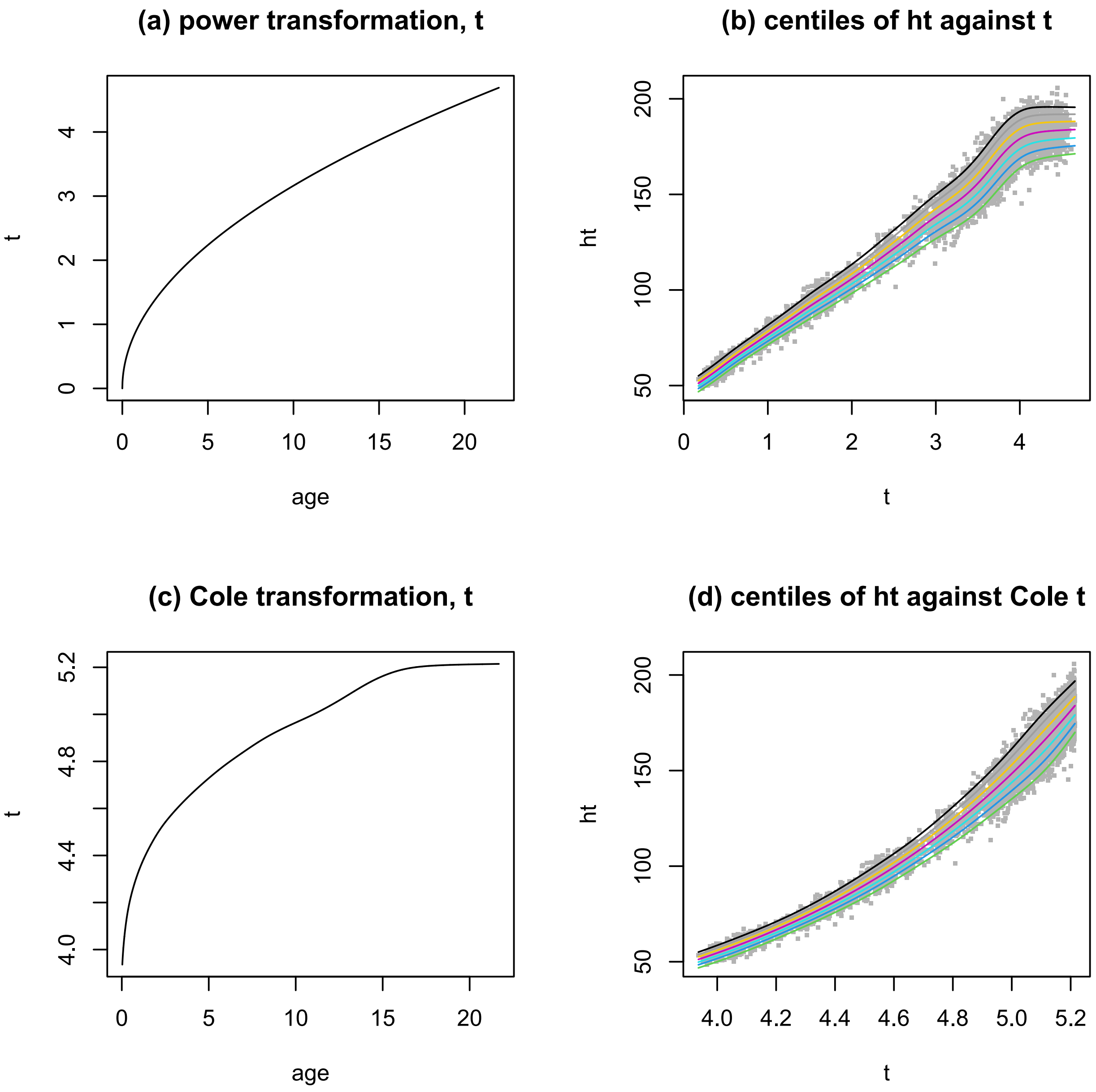

Figure C3: Parameter predictors against age.

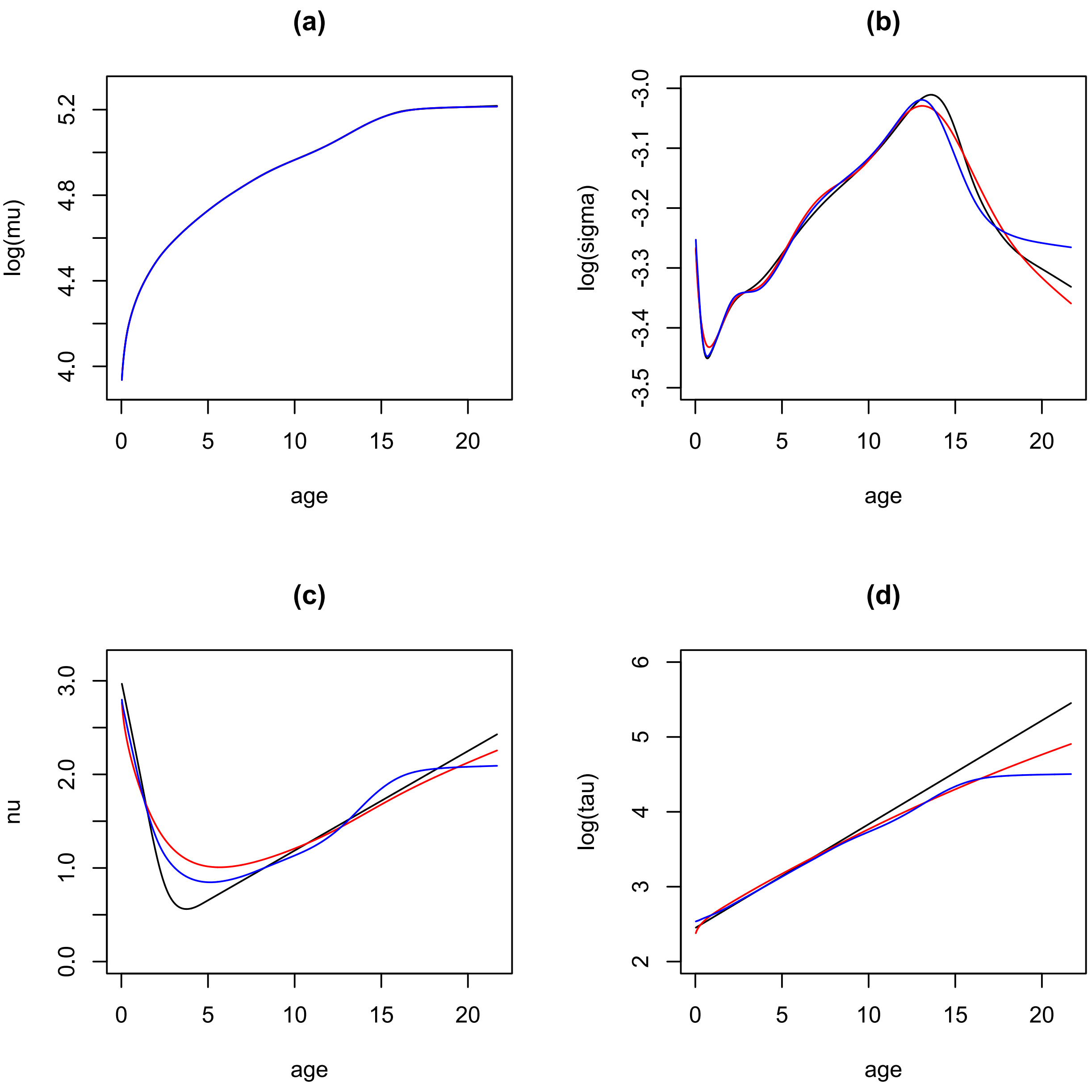

Figure C3 Parameter predictors (a) log(mu), (b) log(sigma), (c) nu, and (d) log(tau), against age. m2: power transform (red), m3: Cole transform (blue), m4:SOP (black).

Figure C4: Z statistics for models m2, m3 and m4

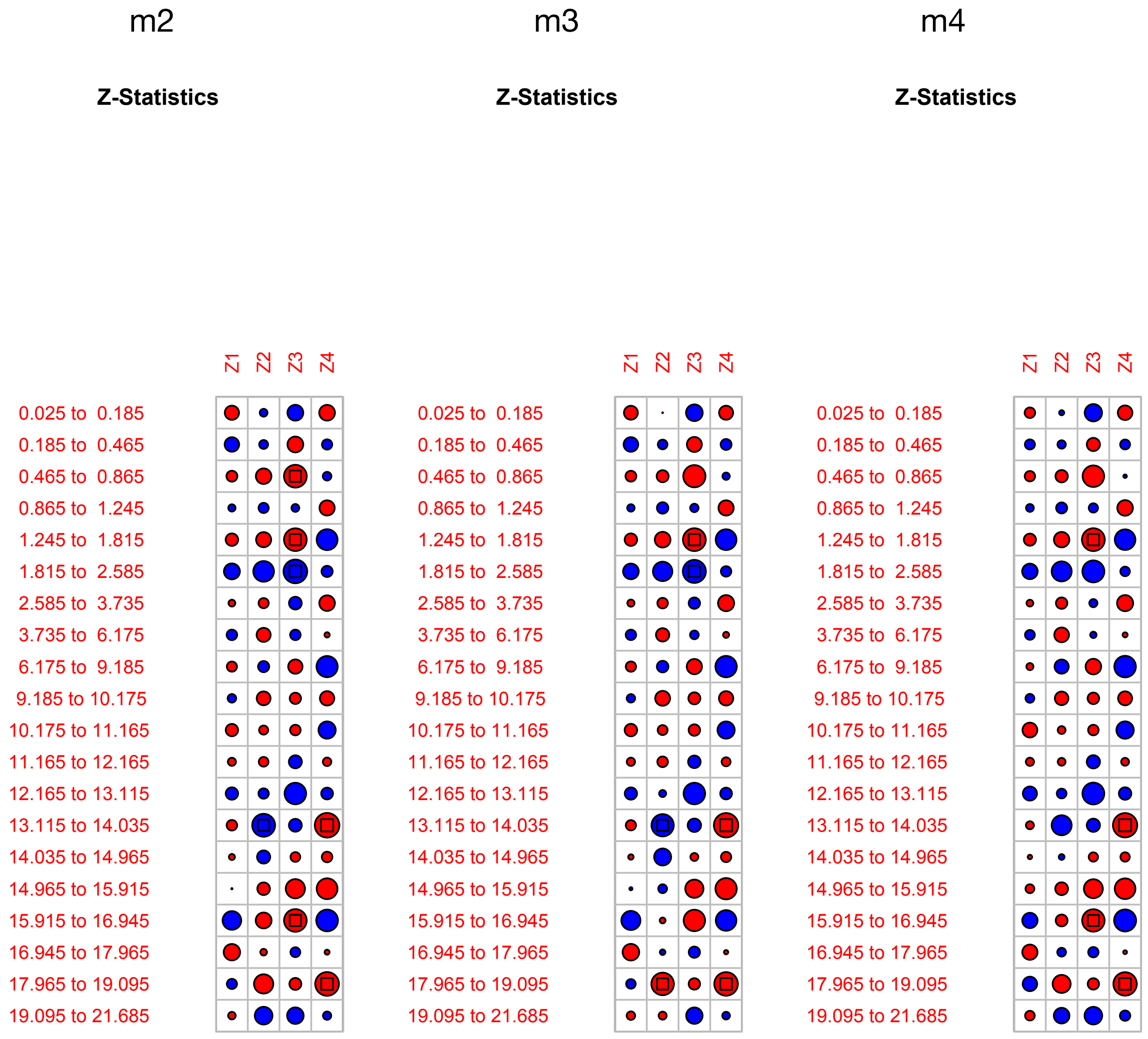

Q and Z statistics

```
Model m2

> round(Q.stats(m2, xvar=dbhh$age,n.inter=20),2)

                         Z1    Z2    Z3    Z4 AgostinoK2    N
 0.025 to  0.185  -0.80  0.24  0.99 -0.98       1.94  362
 0.185 to  0.465   0.83  0.31 -0.99  0.41       1.15  330
 0.465 to  0.865  -0.47 -0.96 -2.05  0.29       4.30  345
 0.865 to  1.245   0.21  0.41  0.24 -0.92       0.90  342
 1.245 to  1.815  -0.62 -0.90 -2.04  1.73       7.16  345
 1.815 to  2.585   1.01  1.69  2.23  0.49       5.21  344
 2.585 to  3.735  -0.18 -0.41  0.64 -0.97       1.34  346
 3.735 to  6.175   0.44 -0.78  0.43 -0.11       0.20  340
 6.175 to  9.185  -0.41  0.49 -0.81  1.78       3.83  346
 9.185 to 10.175   0.29 -0.76 -0.50 -0.82       0.92  347
10.175 to 11.165  -0.60 -0.33 -0.44  1.18       1.58  340
11.165 to 12.165  -0.26 -0.37  0.67 -0.29       0.53  346
12.165 to 13.115   0.61  0.42  1.84  0.55       3.67  348
13.115 to 14.035  -0.44  2.08  0.65 -2.41       6.21  340
14.035 to 14.965  -0.14  0.68 -0.38 -0.44       0.34  344
14.965 to 15.915   0.01 -0.63 -1.46 -1.72       5.08  346
15.915 to 16.945   1.38 -0.99 -2.03  1.87       7.63  341
16.945 to 17.965  -1.06 -0.18  0.38 -0.10       0.16  345
17.965 to 19.095   0.41 -1.47 -0.56 -2.23       5.30  345
19.095 to 21.685  -0.23  1.24  1.08  0.27       1.24  343
TOTAL Q stats      7.72 16.81 29.38 29.31      58.69 6885
df for Q stats     4.93 15.50 16.75 17.52      34.27    0
p-val for Q stats  0.17  0.36  0.03  0.04       0.01    0
```

Model m3

```
> round(Q.stats(m3, xvar=dbhh$age,n.inter=20),2)
```

	Z1	Z2	Z3	Z4	AgostinoK2	N
0.025 to 0.185	-0.76	0.00	1.10	-0.78	1.82	362
0.185 to 0.465	0.84	0.37	-0.89	0.47	1.01	330
0.465 to 0.865	-0.47	-0.57	-1.95	0.21	3.86	345
0.865 to 1.245	0.22	0.52	0.28	-0.92	0.92	342
1.245 to 1.815	-0.62	-0.97	-2.06	1.71	7.18	345
1.815 to 2.585	0.97	1.49	2.11	0.43	4.62	344
2.585 to 3.735	-0.20	-0.44	0.51	-1.03	1.31	346
3.735 to 6.175	0.43	-0.70	0.30	-0.14	0.11	340
6.175 to 9.185	-0.42	0.53	-0.92	1.81	4.13	346
9.185 to 10.175	0.28	-0.88	-0.57	-0.81	0.98	347
10.175 to 11.165	-0.61	-0.39	-0.52	1.17	1.63	340
11.165 to 12.165	-0.27	-0.45	0.62	-0.32	0.49	346
12.165 to 13.115	0.59	0.20	1.83	0.54	3.66	348
13.115 to 14.035	-0.41	1.97	0.71	-2.36	6.09	340
14.035 to 14.965	-0.11	1.14	-0.25	-0.42	0.24	344
14.965 to 15.915	0.04	0.31	-1.32	-1.80	4.98	346
15.915 to 16.945	1.45	-0.13	-1.82	1.68	6.15	341
16.945 to 17.965	-1.09	0.12	0.50	-0.07	0.25	345
17.965 to 19.095	0.36	-2.02	-0.51	-2.20	5.12	345
19.095 to 21.685	-0.29	-0.25	1.06	0.23	1.19	343
TOTAL Q stats	7.84	15.46	27.38	28.35	55.73	6885
df for Q stats	16.45	15.50	16.39	17.15	33.54	0
p-val for Q stats	0.96	0.46	0.04	0.04	0.01	0

Model m4

```
> round(Q.stats(m4, xvar=dbhh$age,n.inter=20),2)

                      Z1    Z2    Z3    Z4 AgostinoK2    N
 0.025 to  0.185  -0.44  0.10  1.19 -0.84       2.13  362
 0.185 to  0.465   0.40  0.28 -0.69  0.39       0.62  330
 0.465 to  0.865  -0.43 -0.60 -1.84  0.05       3.38  345
 0.865 to  1.245   0.24  0.48  0.34 -0.96       1.04  342
 1.245 to  1.815  -0.58 -0.94 -2.07  1.68       7.10  345
 1.815 to  2.585   0.95  1.59  1.91  0.36       3.78  344
 2.585 to  3.735  -0.18 -0.51  0.26 -1.03       1.12  346
 3.735 to  6.175   0.38 -0.86  0.16 -0.10       0.04  340
 6.175 to  9.185  -0.20  0.79 -0.96  1.84       4.30  346
 9.185 to 10.175   0.31 -0.73 -0.53 -0.77       0.88  347
10.175 to 11.165  -0.87 -0.24 -0.44  1.19       1.62  340
11.165 to 12.165  -0.27 -0.24  0.69 -0.24       0.54  346
12.165 to 13.115   0.79  0.37  1.89  0.61       3.94  348
13.115 to 14.035  -0.25  1.58  0.69 -2.35       5.99  340
14.035 to 14.965  -0.08  0.13 -0.34 -0.35       0.24  344
14.965 to 15.915  -0.31 -0.65 -1.42 -1.69       4.88  346
15.915 to 16.945   0.93 -0.56 -1.99  1.92       7.64  341
16.945 to 17.965  -0.92  0.30  0.42 -0.06       0.18  345
17.965 to 19.095   0.83 -1.29 -0.51 -2.17       4.99  345
19.095 to 21.685  -0.37  0.97  1.23  0.42       1.69  343
TOTAL Q stats      6.28 12.44 27.35 28.74      56.09 6885
df for Q stats     3.49 15.29 16.88 18.00      34.88    0
p-val for Q stats  0.13  0.67  0.05  0.05       0.01    0
```

R code for height

```
# This file contains the height R code, 24_05_2025


# First the R code for Supplementary Figures C1, C2, C3 and C4

# Second the R code for selecting the models


####################################################################
```

First the R code for Supplementary Figures C1, C2, C3 and C4

```
# Cole transform from $X$ to $T$ the fitted PREDICTOR of mu
# (since we are using BCTo with a log link for mu)
# with 6 df for smoothing for sigma in m2 and m3 (to avoid overfitting sigma)


####################################################################


rm(list=ls())


# garbage collection
gc()

library(gamlss)

library(devtools)
devtools::install_github("mstasinopoulos/GAMLSS-Additive-terms-2")
library(gamlss.add2)




data(dbhh)
head(dbhh)
dim(dbhh)


####################################################################
```

```
# The choice of distribution between BCCGo, BCTo and BCPEo is given at the end,
# although BCPEo had a slightly lower GAIC(4) than BCTo,
# BCTo had better residuals and so was the chosen distribution

######################################################################
######################################################################
######################################################################
######################################################################

# NOW plot Figure C1

# Figure C1, all the 4 Centile plots for ht:
# Figure C1(a) no transformation
# Figure C1(b) transformation
# Figure C1(c) Cole transformation
# Figure C1(d) SOP

op <- par(mfrow=c(2,2))

######################################################################
######################################################################

# Figure C1(a) no transformation

m1<-
gamlss(ht~pb(age),sigma.fo=~pb(age),nu.fo=~pb(age),tau.fo=~pb(age),family=BCTo,data
=dbhh)

centiles(m1, xvar=dbhh$age,cent=c(3,10,25,50,75,90,97), legend=FALSE, main="(a) no
transform",xlab="age",ylab="ht", cex=0.4)

######################################################################
######################################################################

# Figure C1(b) transformation

# Chosen transformation model for ht

t <- dbhh$age^(1/2)
```

```
dbhh$t <- t

# the sigma model was overfitting, so the sigma df was reduced to
# sigma df=6

m2 <- gamlss(ht~pb(t),sigma.fo=~pb(t, df=6), nu.fo=~pb(t), tau.fo=~pb(t),  family=BCTo,
data=dbhh, n.cyc=200)

centiles(m2, xvar=dbhh$age,cent=c(3,10,25,50,75,90,97), legend=FALSE, main="(b)
power transform",xlab="age",ylab="ht", points=FALSE)

#################################################################
#################################################################

# Figure C1(c) Cole transformation

# Chosen Cole transformation model for ht

# Use the power transformation model m2 to provide a good fitted mu.lp to use
# as the Cole Transformation of age
# CTage = Cole Transformation of age

dbhh$CTage <- m2$mu.lp

# the sigma model was overfitting, so the sigma df was reduced to
# sigma df=6

m3<-gamlss(ht~pb(CTage),sigma.fo=~pb(CTage,
df=6),nu.fo=~pb(CTage),tau.fo=~pb(CTage),
family=BCTo,data=dbhh)

centiles(m3, xvar=dbhh$age,cent=c(3,10,25,50,75,90,97), legend=FALSE, main="(c) Cole
transform",xlab="age",ylab="ht", points=FALSE)

################################################################
################################################################

# Figure C1(d) SOP
```

```
# The SOP model with the lowest GAIC(4) for nseg.sp from 1 to 5
# nseg=50 and nseg.sp=5

nsp <- 5

m4 <- gamlss(ht~SOP(~ ad(age, nseg = 50, nseg.sp = nsp)),
sigma.fo=~SOP(~ ad(age, nseg = 50, nseg.sp = nsp)),
nu.fo=~SOP(~ ad(age, nseg = 50, nseg.sp = nsp)),
tau.fo=~SOP(~ ad(age, nseg = 50, nseg.sp = nsp)),
family=BCTo, data=dbhh)

centiles(m4, xvar=dbhh$age,cent=c(3,10,25,50,75,90,97), legend=FALSE, main="(d)
SOP",xlab="age",ylab="ht", points=FALSE)

par(op)

#######################################################################
#######################################################################
#######################################################################
#######################################################################

# NOW plot Figure C2

op <- par(mfrow=c(2,2))

#######################################################################

# Figure C2(a) Transformation from age to t

agenew <- seq(0,22,0.01)
tnew <- agenew^(1/2)

plot(tnew ~ agenew, type="l",xlab="age",ylab="t",main="(a) power transformation, t")

#######################################################################
```

```
######################################################################

# Figure C2(b)

t <- dbhh$age^(1/2)

dbhh$t <- t


centiles(m2, xvar=dbhh$t,cent=c(3,10,25,50,75,90,97),xlab="t",ylab="ht",main="(b) 
centiles of ht against t", legend=FALSE, cex=0.4)

######################################################################

# par(op)



######################################################################
######################################################################


# Figure C2(c)

# op <- par(mfrow=c(1,2))
CTnew <- m2$mu.lp
plot(CTnew ~ dbhh$age, type="l",xlab="age",ylab="t",main="(c) Cole transformation, t")


######################################################################


# Figure C2(d)

centiles(m3, xvar=dbhh$CTage,cent=c(3,10,25,50,75,90,97),xlab="t",
ylab="ht",legend=FALSE,
main="(d) centiles of ht against Cole t", cex=0.4)

par(op)



######################################################################
######################################################################
######################################################################
######################################################################
```

```
# Figure C3: fitted parameter predictors against age for model m2, m3, and m4


op <- par(mfrow=c(2,2))
plot(dbhh$age,m4$mu.lp,type="l",main="(a)",ylab="log(mu)",xlab="age",ylim=c(3.9,5.3))
lines(dbhh$age,m2$mu.lp,col="red")
lines(dbhh$age,m3$mu.lp,col="blue")
plot(dbhh$age,m4$sigma.lp,type="l",main="(b)",ylab="log(sigma)",xlab="age",ylim=c(-3.5
,-3.0))
lines(dbhh$age,m2$sigma.lp,col="red")
lines(dbhh$age,m3$sigma.lp,col="blue")
plot(dbhh$age,m4$nu.lp,type="l",main="(c)",ylab="nu",xlab="age",ylim=c(0,3.2))
lines(dbhh$age,m2$nu.lp,col="red")
lines(dbhh$age,m3$nu.lp,col="blue")
plot(dbhh$age,m4$tau.lp,type="l",main="(d)",ylab="log(tau)",xlab="age",ylim=c(2,6))
lines(dbhh$age,m2$tau.lp,col="red")
lines(dbhh$age,m3$tau.lp,col="blue")
par(op)


#################################################################
#################################################################
#################################################################
#################################################################


# Figure C4:  Q statistics plots for m2, m3 and m4



op <- par(mfrow=c(1,3))

round(Q.stats(m2, xvar=dbhh$age,n.inter=20),2)
round(Q.stats(m3, xvar=dbhh$age,n.inter=20),2)
round(Q.stats(m4, xvar=dbhh$age,n.inter=20),2)


par(op)



#################################################################
#################################################################
#################################################################
#################################################################



# df's for mu, sigma, nu and tau
```

```
# Deviance and df

GAIC(m1,m2,m3,m4,k=0)


m1$mu.df
m2$mu.df
m3$mu.df
m4$mu.df


m1$sigma.df
m2$sigma.df
m3$sigma.df
m4$sigma.df


m1$nu.df
m2$nu.df
m3$nu.df
m4$nu.df


m1$tau.df
m2$tau.df
m3$tau.df
m4$tau.df



##############################################################################
##############################################################################
##############################################################################
##############################################################################
##############################################################################
##############################################################################
##############################################################################
##############################################################################
```

# Second the R code for selecting the models

```
##############################################################################


# Figure C1(a) no transformation

m1<-
gamlss(ht~pb(age),sigma.fo=~pb(age),nu.fo=~pb(age),tau.fo=~pb(age),family=BCTo,data
=dbhh)
```

```
centiles(m1, xvar=dbhh$age,cent=c(3,10,25,50,75,90,97), legend=FALSE, main="(a) no
transform",xlab="age",ylab="ht", cex=0.4)


######################################################################
######################################################################


# Figure C1(b)  centiles with transformation (with chosen distribution BCTo)
# (NOTE comparison with BCCGo and BCPEo is given at the end of this file)


######################################################################


# Optim search for best power parameter with BCTo and GAIC(4)



dbhh$t <- (dbhh$age)^0.5


k1 <- 4


mbctA <- gamlss(ht~pb(t),sigma.fo=~pb(t), nu.fo=~pb(t), tau.fo=~pb(t),  family=BCTo,
data=dbhh, n.cyc=200)


fnBCT<- function(p)
     {dbhh$t <- dbhh$age^p[1]


mbctN <- gamlss(ht~pb(t),sigma.fo=~pb(t), nu.fo=~pb(t), tau.fo=~pb(t),  family=BCTo,
data=dbhh, n.cyc=200, start.from=mbctA)

     mbctA <- mbctN
   cat("p=", p, " and GAIC=", GAIC(mbctN, k=k1), "\n")
    GAIC(mbctN, k=4)
    }



op1 <- optim(par=c(.5), fnBCT, method="L-BFGS-B", lower=c(.1), upper=c(1),
control = list(maxit = 50, factr=1e10))



op1$par
op1$value
```

```
############################################################################

# Chosen transformation model for ht

t <- dbhh$age^(1/2)

dbhh$t <- t


# the sigma model was overfitting, so the sigma df was reduced to
# sigma df=6


m2 <- gamlss(ht~pb(t),sigma.fo=~pb(t, df=6), nu.fo=~pb(t), tau.fo=~pb(t),  family=BCTo,
data=dbhh, n.cyc=200)

centiles(m2, xvar=dbhh$age,cent=c(3,10,25,50,75,90,97), legend=FALSE, main="(b)
power transform",xlab="age",ylab="ht", points=FALSE)




###########################################################################
###########################################################################



# Figure C1(c) Cole transformation

# Chosen Cole transformation model for ht


# Use the power transformation model m2 to provide a good fitted mu.lp to use
# as the Cole Transformation of age
# CTage = Cole Transformation of age


dbhh$CTage <- m2$mu.lp


# the sigma model was overfitting, so the sigma df was reduced to
# sigma df=6


m3<-gamlss(ht~pb(CTage),sigma.fo=~pb(CTage,
df=6),nu.fo=~pb(CTage),tau.fo=~pb(CTage),
family=BCTo,data=dbhh)
```

```
centiles(m3, xvar=dbhh$age,cent=c(3,10,25,50,75,90,97), legend=FALSE, main="(c) Cole transform",xlab="age",ylab="ht", points=FALSE)



####################################################################
####################################################################
####################################################################
####################################################################


# Figures C1(d) centiles with SOP, chosen distribution BCTo


####################################################################
####################################################################


# The function: find_knot() selects the value of nseg.sp
# which gives the lowest value of GAIC
# Function find_knot() requires the function model() to specify the model



####################################################################

model <- function(nsp = 1, data=dbbmi)
  {
    M<- gamlss(ht~SOP(~ ad(age, nseg=50, nseg.sp = nsp)),
              sigma.fo=~SOP(~ ad(age, nseg=50, nseg.sp = nsp)),
              nu.fo=~SOP(~ ad(age, nseg=50, nseg.sp = nsp)),
              tau.fo=~SOP(~ ad(age, nseg=50, nseg.sp = nsp)),
              family=BCTo, data=dbhh, trace=F, n.cyc=100)
    M
  }
####################################################################

find_knot <- function(from=1, to=10, k=4)
  {
    if(is.null(model)) stop("the model is required")
    models <-  list()
    for (i in from:to)
    {
    assign("nsp", i, envir = .GlobalEnv)
    models[[i]] <- m0 <-  model(i)
    assign("m0",  models[[i]], envir = .GlobalEnv)
    }
    GAICs <- sapply(models, GAIC, k=k)
    GAICs0 <- sapply(models, GAIC, k=0)
```

```
   GAICs2 <- sapply(models, GAIC, k=2)
   GAICs4 <- sapply(models, GAIC, k=4)
   GAICsB <- sapply(models, GAIC, k=log(length(dbbmi$age)))
   pos <- which.min(GAICs)
   out <- list(model=models[[pos]], GAIC=GAICs, min=pos, GAIC0=GAICs0,
GAIC2=GAICs, GAIC4=GAICs4, GAICB=GAICsB)
   out
}
##################################################################
##################################################################
##################################################################


# Selecting the best SOP model with nseg=50

##################################################################
##################################################################

# Fitting the 4th model
######################################################################
########
M <- find_knot(from=1, to=5, k=4)
M
##################################################################

#  model m4
nsp <<- nsp <- which.min(M$GAIC4)

m4  <- gamlss(ht~SOP(~ ad(age, nseg = 50, nseg.sp = nsp)),
sigma.fo=~SOP(~ ad(age, nseg = 50, nseg.sp = nsp)),
nu.fo=~SOP(~ ad(age, nseg = 50, nseg.sp = nsp)),
tau.fo=~SOP(~ ad(age, nseg = 50, nseg.sp = nsp)),
family=BCTo, data=dbhh, n.cyc=100, trace=FALSE)


centiles(m4, xvar=dbhh$age,cent=c(3,10,25,50,75,90,97), legend=FALSE, main="(d)
SOP",xlab="age",ylab="ht", points=FALSE)



##################################################################
##################################################################
##################################################################



# Optim search for best power parameter with BCPEo and GAIC(4)
```

```
dbhh$t <- (dbhh$age)^0.5

k1 <- 4

mbcpeA <- gamlss(ht~pb(t),sigma.fo=~pb(t), nu.fo=~pb(t), tau.fo=~pb(t),  family=BCPEo,
data=dbhh, n.cyc=200)

fnBCPE<- function(p)
     {dbhh$t <- dbhh$age^p[1]

mbcpeN <- gamlss(ht~pb(t),sigma.fo=~pb(t), nu.fo=~pb(t), tau.fo=~pb(t),  family=BCPEo,
data=dbhh, n.cyc=200, start.from=mbcpeA)

     mbcpeA <- mbcpeN
   cat("p=", p, " and GAIC=", GAIC(mbcpeN, k=k1), "\n")
    GAIC(mbcpeN, k=4)
    }

op2 <- optim(par=c(.5), fnBCPE, method="L-BFGS-B", lower=c(.1), upper=c(1),
control = list(maxit = 50, factr=1e10))

op2$par
op2$value

######################################################################

# Optim search for best power parameter with BCCGo and GAIC(4)

dbhh$t <- (dbhh$age)^0.5

k1 <- 4
```

```
mbccgA <- gamlss(ht~pb(t),sigma.fo=~pb(t), nu.fo=~pb(t),  family=BCCGo, data=dbhh,
n.cyc=200)


fnBCCG<- function(p)
     {dbhh$t <- dbhh$age^p[1]


mbccgN <- gamlss(ht~pb(t),sigma.fo=~pb(t), nu.fo=~pb(t), family=BCCGo, data=dbhh,
n.cyc=200, start.from=mbccgA)

     mbccgA <- mbccgN
   cat("p=", p, " and GAIC=", GAIC(mbccgN, k=k1), "\n")
    GAIC(mbccgN, k=4)
    }



op3 <- optim(par=c(.5), fnBCCG, method="L-BFGS-B", lower=c(.1), upper=c(1),
control = list(maxit = 50, factr=1e10))


op3$par
op3$value



######################################################################
######################################################################




# Comparing the power parameter and GAIC values for transformation model
# with the distributions BCTo, BCPEo and BCCGo

op1$par
op1$value

op2$par
op2$value

op3$par
op3$value
```
