# Supplementary material for: Improved Centile Estimation by Transformation And/Or Adaptive Smoothing of the Explanatory Variable
Source: Stat Med. 2026 Feb 5;45(3-5):e70414. doi: 10.1002/sim.70414 (PMC12874224; doi:10.1002/sim.70414)
Supplement: Supplementary file 4 — Data S4. Supporting Information D. [file SIM-45-0-s001.pdf]

Supplementary Materials D

P-splines

When using the P-spline smoothing term pb(x) for a distribution parameter in the gamlss function, then by default it uses penalized cubic B-splines to model the predictor of the distribution parameter. The B-splines are equally spaced on the *X* axis with *nseg* segments, giving (*nseg+3)* B-splines for a cubic piece-wise polynomial.
A quadratic penalty is then applied to these B-spline parameters. The penalty is the sum of squares of second order differences in the sequence of B-spline parameters, multiplied by a constant global smoothing parameter. Hence the same smoothing parameter applies globally to every squared second order difference in the sequence of B-spline parameters.
However P-splines with a global smoothing parameter may be too restrictive and unsuitable when a distribution parameter has high curvature.

Adaptive smoothing in SOP

In the **SOP** adaptive smoothing term ad( ) used in this paper, the global smoothing parameter used in pb( ) is replaced by smoothing parameters that vary locally according to their position in the sequence. Hence they vary with *X*. This is accomplished by specifying a different smoothing parameter for each squared second order difference in the sequence of B-spline parameters, and then *unpenalised* cubic B-splines are used to model the smoothing parameter itself, equally spaced on their sequence position, with *nseg.sp* segments, giving *(nseg.sp+3)* B-splines.
Hence there are *(nseg+3) penalised* B-splines to model the predictor of a distribution parameter, and *(nseg.sp+3) unpenalised* B-splines to model the smoothing parameter itself.
