# Supplementary material for: Improved Centile Estimation by Transformation And/Or Adaptive Smoothing of the Explanatory Variable
Source: Stat Med. 2026 Feb 5;45(3-5):e70414. doi: 10.1002/sim.70414 (PMC12874224; doi:10.1002/sim.70414)
Supplement: Supplementary file 5 — Data S5. Supporting Information E. [file SIM-45-0-s004.pdf]

Supplementary Materials E: Simulation Study

In this section we use simulations to compare the performance of the transformation and adaptive smoothing methods of improved centile estimation. For each of the bmi fitted models m2, m3 and m4 in Section 5.2 (each treated as the 'true' model), we simulated 1000 samples each of size 7294, using the dbbmi values of age and simulating the values of bmi. We then refitted models m1, m2, m3 and m4 from Section\5.2.
In centile estimation the focus is on the accuracy and smoothness of outer centiles which can be used for diagnostic identification of unusually low or high values of the response variable (bmi here) given the corresponding value of the explanatory variable (age here). So we investigated the 3% and 97% outer centiles. The accuracy of the fitted centiles was assessed in the main text of the paper.

To assess smoothness of the centiles we counted the number of turning points and 'bumps' of each refitted centile. A maximum turning point is where the centile increases and then decreases, and a minimum turning point is where the centile decreases and then increases. We define an 'up bump' as where the gradient increases and then decreases, and a 'down bump' as where the gradient decreases and then increases. We then found the mean value of the number of turning points and 'bumps' from the 1000 simulations, for each combination of refitted model and 'true model', for each of the 97% and 3% centiles. The results are given in Tables E1 and E2, for the turning points and bumps, respectively. The conclusion is that the typical refitted model m2 is smoothest, followed by m3, m4 and then m1.

The true number of turning points and 'bumps' for the 'true model' for each of the 97% and3% centiles is also given in the bottom row of Tables E1 and E2. This shows that typically the refitted model m2 has the correct number of turning points, except for the 97% centile of 'true model' m4, while refitted models m3 and m4 often have a higher number of turning points. All the refitted models' 97% and 3% centiles have a higher number of bumps than in the true models, except for the 97% centile of refitted model m2 for true models m3 and m4, which has a lower number of bumps. In conclusion, this suggests that refitted models m3 and m4 may be overfitting, while refitted model m2 may be underfitting true model m4.

We were particularly interested in the presence or absence of the 'kink' in the upper age range in Figure 7(b) (defined as a maximum followed by a minimum turning point) in the fitted σ model. So we counted the number of turning points in σ (above age 6 to avoid counting the presence of a turning point in early years) in each refitted model, in each of the 1000 simulations of each 'true model'. If this was 2 or more it indicated a 'kink' or 'kinks', while if it is 0 or 1 it indicated no 'kink'. We then counted how many of the 1000 simulations had a 'kink' or 'kinks' in σ for each of the 4 refitted modes, for each 'true model'. The results are given in

Table E3. The conclusion is that when the 'true model' has no 'kink' in σ (i.e m2), then refitted models m2, m3 and m4 are likely to have no 'kink', while when the 'true model' has a 'kink' in σ (i.e. models m3 or m4), then refitted models m3 and m4 are likely to have a 'kink' or 'kinks', but refitted model m2 is only likely to have a 'kink' or 'kinks' for true model m4.

In our dbbmi data in Section 5.2, fitted model m2 had no 'kink' in σ, while fitted models m3 and m4 had a 'kink' or 'kinks'.
For 'true models' m2, m3 and m4 the % chance of this occurring was 4.9%, 19.3% and 19.2%, respectively. This suggests that models m3 and m4 are more compatible than m2 with our dbbmi data.

Table E1 Mean number of turning points in the 97% and 3% centiles of the fitted models m1, m2, m3 and m4, from 1000 simulations from each true model m2, m3 and m4. (The last row gives the number of turning points in the true model m2, m3 and m4.)

		97%			3%		
		True			True		
		m2	m3	m4	m2	m3	m4
Fitted	m1	4.9	4.8	5.1	5.5	5.8	5.9
	m2	2.1	2.1	2.2	2.1	2.1	2.2
	m3	2.4	2.9	3.0	2.3	2.4	2.4
	m4	2.8	3.3	3.6	2.6	2.7	2.8
True		2	2	4	2	2	2

Table E2 Mean number of bumps in the 97% and 3% centiles of the fitted models m1, m2, m3 and m4, from 1000 simulations from each true model m2, m3 and m4. (The last row gives the number of bumps in the true model m2, m3 and m4.)

		97%			3%		
		True			True		
		m2	m3	m4	m2	m3	m4
Fitted	m1	14.6	14.6	14.4	14.8	14.8	14.7
	m2	4.4	4.8	5.0	3.7	4.2	4.5
	m3	6.8	7.7	7.5	5.7	5.8	5.7
	m4	7.8	8.1	7.9	6.6	6.6	7.0
True		4	6	7	2	2	4

Table E3 Presence of ‘kink(s)’ in the fitted σ model
for models m1, m2, m3 and m4,
out of 1000 simulations from each true model m2, m3 and m4.
(The last row gives the presence of kinks in the σ of the true model m2, m3 and m4.)

		True		
		m2	m3	m4
Fitted	m1	434	679	877
	m2	94	379	554
	m3	135	560	724
	m4	152	555	772
True		0	1	1
